# Chemical Composition and Biological Activities of Essential Oils of *Curcuma* Species

**DOI:** 10.3390/nu10091196

**Published:** 2018-09-01

**Authors:** Noura S. Dosoky, William N. Setzer

**Affiliations:** 1 Aromatic Plant Research Center, 230 N 1200 E, Suite 102, Lehi, UT 84043, USA; nsdosoky@aromaticplant.org; 2 Department of Chemistry, University of Alabama in Huntsville, Huntsville, AL 35899, USA

**Keywords:** *Curcuma aeruginosa*, *Curcuma glans*, *Curcuma longa*, *Curcuma mangga*, *Curcuma zanthorrhiza*, *Curcuma zedoaria*

## Abstract

Members of the genus *Curcuma* L. have been used in traditional medicine for centuries for treating gastrointestinal disorders, pain, inflammatory conditions, wounds, and for cancer prevention and antiaging, among others. Many of the biological activities of *Curcuma* species can be attributed to nonvolatile curcuminoids, but these plants also produce volatile chemicals. Essential oils, in general, have shown numerous beneficial effects for health maintenance and treatment of diseases. Essential oils from *Curcuma* spp., particularly *C. longa*, have demonstrated various health-related biological activities and several essential oil companies have recently marketed *Curcuma* oils. This review summarizes the volatile components of various *Curcuma* species, the biological activities of *Curcuma* essential oils, and potential safety concerns of *Curcuma* essential oils and their components.

## 1. Introduction

The genus *Curcuma* L. (Zingiberaceae) represents a group of perennial rhizomatous herbs native to tropical and subtropical regions. *Curcuma* is extensively cultivated in tropical and subtropical regions of Asia, Australia, and South America [1]. There are approximately 93–100 accepted *Curcuma* species, however the exact number of species is still controversial [2]. The genus is best known for being an essential source of coloring and flavoring agents in the Asian cuisines, traditional medicines, spices, dyes, perfumes, cosmetics, and ornamental plants [3]. Several *Curcuma* species are used medicinally in Bangladesh, Malaysia, India, Nepal, and Thailand [4] for treating pneumonia, bronchial complaints, leucorrhea, diarrhea, dysentery, infectious wounds or abscesses, and insect bites [2,4,5]. The rhizome is the most commonly used part of the plant. The main active components of the rhizome are the nonvolatile curcuminoids and the volatile oil [6,7,8]. Curcuminoids (curcumin, demethoxycurcumin, and bisdemethoxycurcumin) are nontoxic polyphenolic derivatives of curcumin that exert a wide range of biological activities [9]. Several phytochemical studies on *Curcuma* oils led to the identification of sesquiterpenoids and monoterpenoids as the major components [9]. The essential oil (EO) of *Curcuma* species possesses a wide variety of pharmacological properties, including anti-inflammatory, anticancerous, antiproliferative, hypocholesterolemic, antidiabetic, antihepatotoxic, antidiarrheal, carminative, diuretic, antirheumatic, hypotensive, antioxidant, antimicrobial, antiviral, insecticidal, larvicidal, antivenomous, antithrombotic, antityrosinase, and cyclooxygenase-1 (COX-1) inhibitory activities, among others [7,10,11,12,13,14,15,16,17]. *Curcuma* oils are also known to enhance immune function, promote blood circulation, accelerate toxin elimination, and stimulate digestion [18,19]. *C. longa* (turmeric) and *C. zedoaria* (zedoary) are the most extensively studied species of *Curcuma* due to their high commercial value. Other *Curcuma* species have been studied to a lesser degree. This review provides an update on recent studies performed on the chemical composition and biological studies on genus *Curcuma*. The search engines Google Scholar, PubMed, ScienceDirect, and ResearchGate were used to access the literature.

## 2. Volatile Components of *Curcuma* spp.

**Figure 1:**
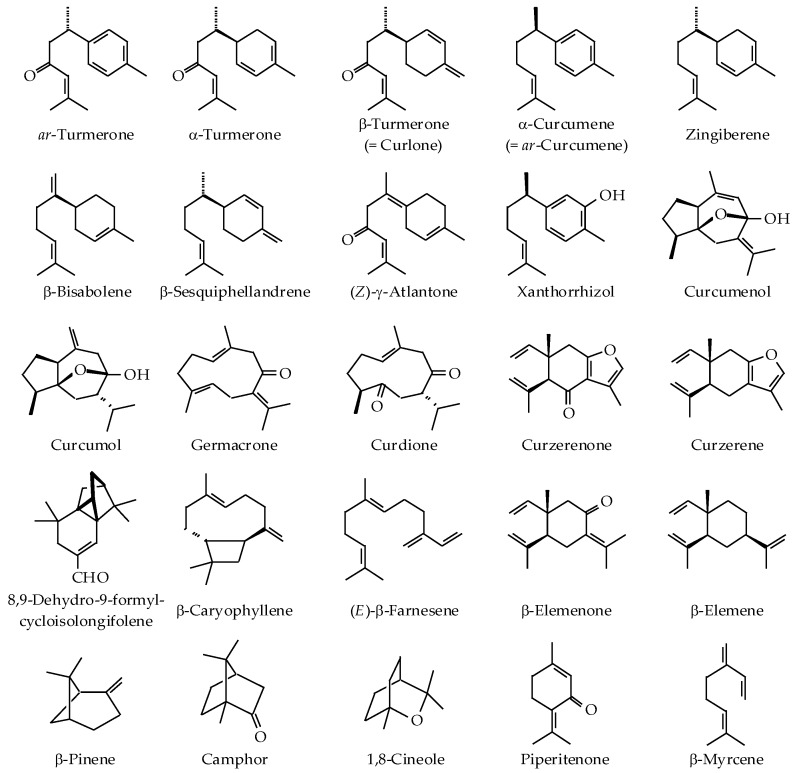
Chemical structures of key volatile components in the essential oil from *Curcuma* spp. rhizomes.

Generally, essential oils of *Curcuma* species are obtained by hydro- or steam distillation of the fresh or dry rhizome [20]. Alternatively, *Curcuma* volatiles have also been obtained by solvent extraction or supercritical fluid extraction of the powdered rhizome [21]. More recently, solid-phase microextraction (SPME) has been employed as a solvent-free technique to capture and concentrate volatiles from different plant parts. Industrially, *Curcuma* oil is produced during oleoresin processing as a byproduct of curcumin extraction [22]. After curcumin is isolated from the oleoresin, the mother liquor (about 70–80%) is known as “curcumin-removed turmeric oleoresin” (CRTO) [22]. The oil is then extracted from CRTO by hexane or other organic solvent, a process that could result in the loss of the highly volatile components during solvent evaporation [21]. The use of alcohols as the solvent for oil extraction might cause esterification, etherification, and acetal formation [21]. The volatile components of different *Curcuma* species, typically identified by gas chromatography mass spectrometry, are summarized in Table 1. In general, *Curcuma* species produce a wide variety of volatile sesquiterpenes, monoterpenes, and other aromatic compounds [17,23]. The chemical structures of key volatile components are presented in Figure 1. There is a tremendous variation in the composition of *Curcuma* essential oils (EOs). Differences in the oil chemical profile might be due to genotype, variety, differential geography, climate, season, cultivation practices, fertilizer application, stress during growth or maturity, harvesting time, stage of maturity, storage, extraction, and analysis methods [24,25,26,27]. However, some of the variation could be due to misidentification of the plant species or some of the components.

### 2.1. Curcuma longa L.

*Curcuma longa* (syn. *C. domestica* Valeton and *C. brog* Valeton) is also known as “turmeric” worldwide, “kurkum” in Arabic, and “haldi” in Hindi and Urdu. Turmeric is cultivated extensively worldwide but is native to Southeast Asia [76]. It is a perennial herb grown on a very large scale in India, Pakistan, Bangladesh, China, Taiwan, Thailand, Sri Lanka, East Indies, Burma, Indonesia, and Northern Australia [66]. In the West, it is produced in Costa Rica, Haiti, Jamaica, Peru, and Brazil [116]. Turmeric is commercially available as a whole rhizome (fresh, dried, and cured by cooking in water, drying in shade, and polishing), turmeric powder, extracts, and oleoresins, with the powder being the most commonly consumed form. India is the largest producer and consumer of turmeric [66,117,118]. The plant is famous for its culinary and medicinal uses. Turmeric is the golden spice that gives many Asian dishes their yellow color and pungent earthy flavor. It is an essential ingredient of curry powders, accounting for about 10–30% of the blend [119]. In traditional medicine, turmeric is extensively used as a carminative, digestive aid, stomachic, appetizer, anthelmintic, tonic and laxative [120]. It is also used for treating fever, gastritis, dysentery, infections, chest congestion, cough, hypercholesterolemia, hypertension, rheumatoid arthritis, jaundice, liver and gall bladder problems, urinary tract infections, skin diseases, diabetic wounds, and menstrual discomfort [66,94,121]. Turmeric is used in many religious rituals, as a dye, and as a cosmetic [122,123]. Turmeric rhizome typically contains carbohydrates (69.4%), protein (6.3%), fat (5.1%), and minerals (3.5%) [124].

Turmeric oleoresin is an orange-red viscous liquid, prepared from the powdered rhizome by solvent extraction with a yield of about 12% [119]. The main active components in the rhizome are essential oil and curcuminoids. The volatile oil is responsible for the turmeric aroma, while the curcuminoids (curcumin and its analogues) are responsible for its bright yellow color [65,119]. It is worth mentioning that curcumin, present in turmeric rhizomes, oleoresin, and CO_2_ extract, has not been reported in the essential oil [125]. Turmeric chemotypes in the literature vary widely. Hundreds of compounds have been identified from the turmeric EO; however, the major constituents are *ar*-turmerone, α-turmerone, and β-turmerone, followed by notable amounts of α-zingiberene, curlone, *ar*-curcumene, α-santalene, santalenone, β-sesquiphellandrene, (*Z*)-β-ocimene, β-bisabolene, β-caryophyllene, α-phellandrene, (*Z*)-β-farnesene, humulene oxide, β-selinene, caryophyllene oxide, (*E*)-γ-atlantone, 1,8-cineole, and terpinolene. Samples from Brazil had (*Z*)-γ-atlantone, *ar*-turmerone, and (*E*)-γ-atlantone [78]. A sample from north-central Nigeria was a mixture of β-bisabolene, (*E*)-β-ocimene, β-myrcene, 1,8-cineole, α-thujene, α-phellandrene, limonene, zingiberene, and β-sesquiphellandrene [85]. Turmeric EOs from Sri Lanka and São Tomé and Principe had α-phellandrene, α-turmerone, 1,8-cineole, *p*-cymene, *ar*-turmerone, β-turmerone, and terpinolene as the main constituents [13,87]. Some turmeric rhizome EOs from India contained 1,8-cineole, α-turmerone, β-caryophyllene, β-elemene, *ar*-turmerone, β-sesquiphellandrene, camphor, α-farnesene, and (*Z*, *Z*)-farnesol [17,86]. Wide variations are also found between the EO obtained from fresh and dry rhizomes. Unfortunately, comparative data on the chemical composition of volatile oil from fresh and dry rhizomes from a single source, single geographical location, and same season are scarce. However, some of the variation could be explained by the rhizome processing. The order of yield is cured > fresh > dried rhizome [67]. Some of the highly volatile low-boiling-point compounds might be lost during rhizome processing that involves grating, heating, drying, and grinding [126]. For example, turmerones are major components in fresh rhizomes, while only minor ones in dry rhizomes, which might be due to oxidation/polymerization of the two conjugated double bonds [23].

Turmeric root EO from Kerala, India contained *ar*-turmerone (46.8%) and *ar*-curcumene (7.0%) as the main components [70]. There are seven different chemotypes of the *C. longa* leaf EO reported so far [94]: (1) *ar*-turmerone-rich chemotype [94]; (2) α-phellandrene-rich chemotype [66,68,70,83,89,91,95,96]; (3) terpinolene-rich chemotype [84,86,93]; (4) β-sesquiphellandrene-rich chemotype [92]; (5) *p*-cymene-rich chemotype [90]; (6) 1,8-cineole-rich chemotype [127]; and (7) myrcene-rich chemotype [128]. Turmeric flower EO from India contained *p*-cymen-8-ol (26.0%) and terpinolene (7.4%) [70], while the floral oil from France had terpinolene (67.4%) as the main component [84].

### 2.2. Curcuma zedoaria (Christm.) Roscoe

*Curcuma zedoaria* (syn. *C. malabarica* Velay, Amalraj and Mural; *C. raktakanta* Mangaly and M. Sabu) is commonly known as “zedoary” and “white turmeric” in English and “er-jyur” in Chinese. It is native to northeast India and Indonesia [112], but widely cultivated in subtropical regions including India, Southeast Asia, Thailand, Indonesia, Japan, and China [109]. Zedoary rhizome looks like ginger from the outside (wrinkled gray, ash-colored) and like turmeric from the inside (brownish red-yellow). It has a less intense aroma that can be rated between turmeric and mango. In addition, the rhizome powder of *C. zedoaria* is used for culinary purposes because of its unique smell, but has a very bitter and pungent taste, causing many people to substitute it with ginger. Different parts of *C. zedoaria* have been used for treating hematologic and circulation abnormalities [8], wounds, digestive problems, flatulence, skin diseases, and various infections [129]. Zedoary rhizome extracts exhibit anticancer [130], anti-inflammatory [131], analgesic [132], antiallergic [133], antiparasitic against *Entamoeba histolytica* [134], antibacterial and antifungal activities [16]. *C. zedoaria* rhizome oil is mainly composed of sesquiterpenoids (80–85%) and monoterpenoids (15–20%). The reported major components of *C. zedoaria* rhizome EO include epicurzerene (19.0–46.6%), curzerene (10.4%), curdione (7.0–19.6%), [7,109,110], curzerenone (22.3–31.6%) [111,112], debromofiliforminol (31.5%) [13], 1,8-cineole (18.5–40.8%), β-sesquiphellandrene (21.5%), *p*-cymene (18.4%), curcumenene (18.7%), and α-phellandrene (14.9%) [17,37]. α-Terpinyl acetate (8.4%), isoborneol (7.0%), dehydrocurdione (9.0%) and selina-4(15),7(11)-dien-8-one (9.4%) are the main components in the leaf oil [115].

### 2.3. Curcuma aeruginosa Roxb.

*Curcuma aeruginosa* is also known as “pink-and-blue ginger” or “black curcuma” in English, “temu hitam” in Malaysia, and “waan-maha-mek”, “kamindam”, or “kajeawdang” in Thailand [2,135]. It is an aromatic perennial herb (30–40 cm high) that is thought to have been derived from Burma and spread to tropical countries like Malaysia, Thailand, India, and Indonesia [29]. *C. aeruginosa* has a distinctive ginger-like odor [43]. In folk medicine, *C. aeruginosa* rhizome is used for treating dyspepsia, gastritis, dysentery, flatulence, diarrhea, postpartum problems, and parasitic infections [28,43,136,137,138,139], as well as tumors, bronchitis, and asthma [140]. *C. aeruginosa* EO is usually composed of relatively equal amounts of monoterpenes and sesquiterpenes. The rhizome EO of *C. aeruginosa* is mainly composed of 8,9-dehydro-9-formyl-cycloisolongifolene (35.3%), dihydrocostunolide (22.5%) [28], germacrone (23.5%), curzerenone (11.8%) [29], dehydrocurdione (27.6%), curcumenol (15.1%), 1,8-cineole (22.7%), germacrone (17.7%) [30], camphor (29.4%), germacrone (21.2%) [2], curcumenol (38.7%), and β-pinene (27.5%) [17]. The leaf oil is made of 1,8-cineole, curzerenone, furanogermenone, camphor, and furanodienone [34] or curzerene, germacrone, 1,8-cineole, and camphor [35].

### 2.4. Curcuma zanthorrhiza Roxb.

*Curcuma zanthorrhiza* (Syn. *C. xanthorrhiza*), also known as “wan-salika-linthong” in Thailand, and “temulawak”, “Javanese ginger”, or “Javanese turmeric” in Indonesia [108], is native to Indonesia and the Malay Peninsula and is cultivated in Thailand, Philippines, Malaysia, and Sri Lanka [141]. In Indonesia, *C. zanthorrhiza* rhizomes are utilized as food coloring, a spice, a source of starch, a dye, in cosmetics, and in traditional medicine [108]. In traditional medicine, infusions and extracts of *C. zanthorrhiza* rhizome are used in treating hypertension, diabetes, constipation, fevers, diarrhea, dysentery, liver damage, gastric problems, rheumatism, haemorrhoids, skin eruptions, and some cancers [108,141,142,143]. The fresh rhizome or dried powder of *C. zanthorrhiza* is used for skin diseases in northern Thailand [136]. Generally, monoterpenes predominate (80–88%) the rhizome EO of *C. zanthorrhiza* [2]. Three major chemotypes can be observed: (1) α-curcumene-dominated chemotype [108]; (2) α-terpinolene-rich chemotype [2]; and (3) xanthorrhizol-dominated chemotype [31,71]. Xanthorrhizol accounts for 64.4% of the hydrodistilled oil from fresh *C. zanthorrhiza* rhizome from India [144], while only 8.0% of the oil obtained by supercritical carbon dioxide extraction [145]. The hexane extract of *C. zanthorrhiza* gave α-curcumene, germacrone, and zederone, whereas the dichloromethane extract contains curcumin and xanthorrhizol [146].

### 2.5. Curcuma aromatica Salisb.

*Curcuma aromatica* (syn. *C. wenyujin* Y.H. Chen and C. Ling), commonly known as wild turmeric, is a perennial plant that grows in the tropical and subtropical regions. It is broadly cultivated in China, India, and Japan [47]. It is used as a flavoring and coloring agent as well as a traditional medicine for eliminating blood stasis, slowing the aging process, alleviating pain, and protecting against liver diseases [54,147]. *C. aromatica* is used to promote blood circulation [148] and to fight various microbial infections [149]. Internally, wild-turmeric rhizomes are used as a tonic and carminative, while externally they are applied for treating skin eruptions and infections, and to improve complexion, ease bruises, and relieve sprains and snake bites [150]. It possesses anti-inflammatory, anticancer, antiangiogenic, antioxidative, and antimicrobial activities [54,151]. It is known to produce antidepressant-like effects in chronic unpredictable stress-induced depression [152]. The major constituents in *C. aromatica* rhizome EO contain 8,9-dehydro-9- formyl-cycloisolongifolene (2.7–36.8%), germacrone (4.3–16.5%), *ar*-turmerone (2.5–17.7%), turmerone (2.6–18.4%) [47], curdione (50.6%) [49], camphor (18.8–32.3%) [13,17,46,48], xanthorrhizol (26.3%), *ar*-curcumene (19.5%), di-*epi*-α-cedrene (16.5%) [51], curcumol (35.8%), and 1,8-cineole (12.2%) [50]. The leaf EO contains camphor (24.0%–28.5%) and *p*-cymene (25.2%) as the main components [37,46,54].

### 2.6. Curcuma phaeocaulis Valeton

*Curcuma phaeocaulis* is known as “pengezhu”, “ezhu” and “heihejianghuang” in Chinese [153]. *C. phaeocaulis* is widely found throughout the southern parts of China [154]. In Chinese medicine, the rhizome of *C. phaeocaulis* is one of the commonly prescribed herbs, and is known as *Rhizoma Curcumae*. Individually or in combination with other herbs, *Rhizoma Curcumae* is used in controlling gastritis, reducing blood stasis, and alleviating pain [100]. The State Food and Drug Administration of China has already approved *Rhizoma Curcumae* oil as a therapeutic remedy for several disorders [100]. *Rhizoma Curcumae* preparations and oils possess several pharmacological activities, including analgesic, hepatoprotective, antithrombic, antimicrobial, antiviral, and anti-inflammatory effects [155]. *C. phaeocaulis* rhizome EO has 8,9-dehydro-9-formyl-cycloisolongifolene (15.6–46.2%), germacrone (8.9–21.2%), and curlone (0.8–20.2%) as the main constituents [100].

### 2.7. Curcuma amada Roxb.

*Curcuma amada* is a perennial herb native to East India. It is commonly known as the “mango ginger” and “manga manjal” because of its raw mango flavor that is mainly attributed to the presence of δ-3-carene, myrcene, and (*Z*)-β-ocimene [156]. It is used in culinary preparations, medicines, and as a source of starch [157]. In the Ayurveda and Unani medicinal systems, mango ginger is used as an appetizer, laxative, diuretic, antipyretic, aphrodisiac, emollient, and expectorant. It also helps in treating bronchitis, asthma, itching, inflammation, and skin diseases [157]. A paste made from the rhizome is used externally to relieve bruises, sprains, contusions, and rheumatic pain. The rhizome EO of *C. amada* from India is dominated by myrcene [37,38,39]. A totally different composition for the *C. amada* rhizome EO was reported by Mustafa et al. [40], with (*Z*)-β-farnesene (21.9%), 6,9-guaiadiene (19.8%), α-longipinene (14.8%), and α-guaiene (14.5%), and camphor (5.5%) as the major constituents and thymol (4.9%) as the aromatic constituent contributing to the odor of the oil. Other reported compositions include (*E*)-hydroocimene, (*Z*)-hydroocimene, myrcene, and linalool [41], and *ar*-curcumene, β-curcumene, camphor, curzerenone, and 1,8-cineole [42]. The leaf oil is made of camphor, *epi*-curzerenone, curzerenone, and isoborneol [38].

### 2.8. Curcuma caesia Roxb.

*Curcuma caesia* is commonly known as “black turmeric” in India due to the dark bluish color of its rhizome. It grows wild in some parts of India, Malaysia, Thailand, and Indonesia. Leaves and rhizomes of black turmeric are used in traditional medicine. *C. caesia* rhizome is aromatic, carminative, and a stimulant. A paste of the rhizome is used for treating dysentery and as poultice in rheumatic pain, sprains, and bruises. When applied externally, black turmeric is used in India to alleviate toothaches, treat skin and wound infections, and cure rheumatism. Chewing small amounts of the rhizomes is used to relieve digestive problems and kidney disorders; however, excessive intake of black turmeric may lead to vomiting [155]. The rhizome EO of *C. caesia* from south India was composed mainly of 1,8-cineole (30.1%) followed by camphor, *ar*-curcumene, and camphene [17], while the oil from central India has camphor (28.3%), followed by *ar*-turmerone, (*Z*)-β-ocimene, *ar*-curcumene, and 1,8-cineole [57]. The leaf EO is made of 1,8-cineole (27.0%) and camphor (16.8%) [58].

### 2.9. Other Curcuma Species

Other *Curcuma* species have been investigated to a lesser degree, in part due to their limited commercial interest. *Curcuma albiflora* Thwaites rhizome EO contains α-pinene, caryophyllene oxide, and alconfor [13]. *C. alismatifolia* Gagnep, commonly known as Siam tulip, is an ornamental plant. (–)-Xanthorrhizol (52.4%) and *ar*-curcumene (27.4%) dominate the root EO, while β-curcumene (42.0%), (–)-xanthorrhixol (36.6%), and α-curcumene (7.5%) are the major components of the rhizome EO [36]. *C. angustifolia* Roxb. rhizome is used in folk medicine to treat asthma, dysentery, fungal infections, fevers, as well as an analgesic, antiparasitic, and muscle relaxant [5,158,159,160]. The EO obtained from *C. angustifolia* root is dominated by β-elemenone (65.0%) [44], while the rhizome EO has three chemotypes so far: (1) xanthorrhizol isomer- methyleugenol-rich chemotype [43]; (2) germacrone and camphor-rich chemotype [43]; and (3) curzerenone-dominated chemotype [45] as the main components. In the fresh rhizome EO of *C. aurantiaca* Zijp, piperitenone accounts for 65.2% [55]. Another sample of *C. aurantiaca* EO from India was made of 1,8-cineole, camphor, germacrone, β-elemene, curzerene, and β-elemenone [56]. *C. elata* Roxb. rhizome EO from China is mainly made of 8,9-dehydro-9-formyl- cycloisolongifolene (52.2%), followed by germacrone (14.0%) [49]. The rhizome of *C. glans* K. Larsen and J. Mood has been traditionally used in treating tonsillitis, sore throat, wounds or abscesses in the mouth, throat, and nose, as well as the herpes simplex virus [2,136]. Sesquiterpenes (50.10%) dominates the EO of *C. glans* rhizome. The Thai oil of *C. glans* rhizome is dominated by germacrone, camphor, β-pinene, and 2-nonanol [2]. The rhizome oil of *C. haritha* Mangaly and M. Sabu contains camphor, 1,8-cineole, isoborneol, curdione and camphene as the main constituents [59]. Germacrone is the main component in the rhizome EO of *C. harmadii* Gagnep [60] and *C. leucorhiza* Roxb. [161]. *C. harmadii* EO from Vietnam has 1,8-cineole, germacrone, β-pinene, β-elemene, and isocurcumenol [60]. The major constituents of *C. inodora* Blatt rhizome EO are curzerenone, germacrone, curdione, and 1,8-cineole [61]. The oil obtained from *C. kwangsiensis* S.G. Lee and C.F. Liang fresh rhizome was made of α-elemene, germacrene D, spathulenol, curdinone, and β-bisabolene [49]. The major volatile components of *C. kwangsiensis* from Guangxi, China include germacrone, β-elemenone, β-elemene, curzerenone, and curdione [62].

*Curcuma mangga* Valeton and Zijp. rhizome EO was reported to have two chemotypes, (1) caryophyllene oxide and caryophyllene-rich chemotype [28], and myrcene-dominated chemotype [31,98,99]. The major components of *C. nankunshanensis* N. Liu, X.B. Ye and Juan Chen fresh rhizome EO from China were curdione, germacrone, 8,9-dehydro-9-formyl-cycloisolongifolene, and velleral [49]. Caryophyllene, phytol, humulene, elemene, and caryophyllene oxide were detected as major compounds in the EO of the *C. oligantha* Trimen rhizome [13]. *C. pseudomontana* J. Graham rhizome EO from India was made of β-elemenone, pseudocumenol, germacrone, 2-(4-methoxyphenyl) *N*, *N*-trimethyl-1-pyrrolamine, and (1,5-dimethyl-4-hexenyl)-4-methylbenzene [102]. The powdered rhizome of *C. purpurascens* Blume, also known as “temu tis” in Indonesia, is taken in combination with other herbs to treat cough and skin infections. The EO of *C. purpurascens* rhizome contains turmerone as the major constituent, followed by germacrone, *ar*-turmerone, germacrene B, curlone, and curzerene [103]. *C. rhabdota* Sirirugsa and M.F. Newman contains germacrone, butyl butanoate, *sec*-butyl butanoate, camphene, and germacrene B as the main constituents [104]. *C. rubescens* Roxb. rhizome EO from China was composed of zerumbone, *ar*-turmerone, germacrone, camphor, and aromadendrene oxide [49]. *C. sichuanensis* X.X. Chen rhizome EO from China was made of germacrone followed by β-elemenone and isoaromadendrene epoxide [49]. Samples from Sichuan, China showed two more different compositions [50,105]. *C. singularis* Gagnep. fresh rhizome EO contained camphor and germacrone [106]. *C. sylvatica* Valeton rhizome oil from India was dominated by α-fenchene [17]. *C. trichosantha* Gagnep EO was mainly made of curdione [107]. *C. yunnanensis* N. Liu and S.J. Chen rhizome EO from China was composed of germacrone, 8,9-dehydro-9-formyl-cycloisolongifolene, dihydrocostunolide, β-farnesene, and aromadendrene oxide [49]. To the best of our knowledge, there are no published studies on the other *Curcuma* species.

## 3. Biological Activities of *Curcuma* Oils

Members of Zingiberaceae are known for containing terpenoids, flavonoids, phenypropanoids and sesquiterpenes, which have antitumor activities [110,162]. Some *Curcuma* essential oils have remarkable antioxidant and antimicrobial activities that make them ideal candidates for use in pharmaceutical and cosmetic industries. The variations in chemical composition imply the possibility of different biological activities of the same plant species from different locations. A summary of the biological activities of different *Curcuma* essential oils is presented in Table 2.

### 3.1. Turmeric (C. longa) Essential Oil

Turmeric EO has the potential to provide protection against cardiovascular diseases. The oil was reported to have antihyperlipidemic effects on high-fat diet (HFD)-induced hyperlipidemia in rats [75]. It markedly decreased the levels of triglycerides, free fatty acids, total cholesterol in serum, and low-density lipoprotein (LDL) cholesterol, while increasing the level of high-density lipoprotein (HDL) cholesterol. Turmeric EO also showed antihyperlipidemic effects in hyperlipidemic golden Syrian hamsters via reducing lipid-induced oxidative stress, platelet activation, and vascular dysfunction [163]. Chronic dietary supplementation of turmeric EO (≥620 mg/kg/day) showed antidiabetic and hypoglycemic effects in diabetic mice by normalizing serum glucose [164]. Ingestion of turmeric oleoresin and essential oil inhibited both the increase in blood glucose and the development of abdominal fat mass in obese diabetic rats [165]. Turmeric EO also inhibited α-glucosidase and α-amylase activities in a dose-dependent manner due to the presence of *ar*-turmerone [96,166,167].

In addition, the oil showed remarkable antioxidant activity as judged by 1,1-diphenyl-2-picrylhydrazyl (DPPH) radical scavenging activity assay, ferric reducing/antioxidant power (FRAP) assay, superoxide anion radical scavenging activity assay, and metal-chelating activity assay [50,74,168,169]. Turmeric EO prevented oxidative stress in *Brycon amazonicus* via reducing the synthesis or release of cortisol and increasing the activity of antioxidant enzymes, and thereby protecting from the formation of reactive oxygen species excess [23,67,96]. The potent antioxidant activity of turmeric EO is thought to be responsible for inhibiting brain-edema formation, one of the most dangerous consequences of ischemic brain injury [170]. Treatment with turmeric EO reduced nitric oxide production derived by inducible nitric oxide synthase (iNOS) during ischemic injury [231]. Turmeric EO inhibited copper-mediated oxidation of LDL in the thiobarbituric acid reactive substances assay (IC_50_ = 7.8 ± 0.2 µg/mL) [71]. Turmeric EO (250–500 mg/kg p.o. or i.p.) showed neuroprotective effects in rat embolic-stroke model [170,171]. In filament model of middle cerebral-artery occlusion, pretreatment with turmeric EO showed a neuroprotective effect by inhibiting the generation of free radicals [170,171]. Its neuroprotective efficacy was mediated by reducing endothelial cell-mediated inflammation in postmyocardial ischemia/reperfusion in rats [166,172]. It was also suggested that the ability of the oil to access the brain after stroke was via the transcellular lipophilic pathway [170]. Turmeric EO (500 mg/kg, p.o.) was an efficacious and safe antiplatelet agent [174] and was protective against intravascular thrombosis in myocardial ischemia-reperfusion and thrombosis rat models [172,173]. Turmeric oil was effective in treating some respiratory disorders by preventing asthma, removing sputum, and relieving cough [232]. The oil was reported to have anticancer and anti-inflammatory effects [176,178]. It was active against human mouth epidermal carcinoma (KB) cells and mouse leukemia (P388) cells, with respective IC_50_ values of 1.088 and 0.084 mg/mL [177]. It was also cytotoxic to the pancreatic cancer (PANC-1), melanoma (B16), prostate cancer (LNCaP), and human cervical adenocarcinoma (HeLa) cell lines due to the presence of *ar*-turmerone, α-turmerone, β-turmerone, curlone, *ar*-curcumene, zingiberene, and β-sesquiphellandrene [23,74,175,176]. Crude organic extracts of turmeric-inhibited lipopolysaccharide (LPS)-induced production of tumor necrosis factor (TNF)-α (IC_50_ = 15.2 μg/mL) and prostaglandin E2 (PGE2; IC_50_ = 0.92μg/mL) in human leukemia (HL-60) cells [181]. In combination with curcumin, turmerones from turmeric EO abolished inflammation-associated mouse-colon carcinogenesis [233]. Turmeric EO demonstrated strong protective effect against benzo[a]pyrene-induced increase in micronuclei in circulating lymphocytes and protected against cytogenetic damage in patients suffering from oral submucous fibrosis, a precancerous condition for oral cancer [179,180].

Moreover, turmeric EO showed potent antiarthritic and joint protective effects on an animal model of rheumatoid arthritis [23,182]. As a result of treatment with crude or refined turmeric oil (i.p.), joint swelling was dramatically inhibited (90–100% inhibition) in female rats with streptococcal cell wall-induced arthritis [182]. Turmeric EO was reported to have antihepatotoxic [23,183], antiatherosclerotic [96], hypothermic, anxiolytic, sedative, anticonvulsant [81], and spasmolytic [185] activities. Turmeric EO protected against accelerated atherosclerosis, inflammation, and macrophage foam-cell formation induced by arterial injury through modulating the genes involved in plaque stability, lipid homeostasis, and inflammation [184]. Turmeric EO (200 mg/kg) exhibited antifatty liver and hepatoprotective activities in acute ethanol-induced fatty liver in rats through decreasing the activities of serum enzymes and levels of serum triglyceride, serum total cholesterol, and hepatic malondialdehyde, while restoring the level of reduced glutathione as well as the activities of glutathione-*S*-transferase and superoxide dismutase [186]. The oil was markedly antimutagenic against sodium azide in the Ames test [178,187]. Turmeric oil showed remarkable sedative and anesthetic effects in mice [81] and fish [96] in different experimental protocols. Interestingly, *ar*-turmerone isolated from turmeric EO is a potent antivenom against snakebites. It neutralized both the hemorrhagic activity present in *Bothrops jararaca* venom, and the lethal effect of *Crotalus durissus* venom in mice [188].

Additionally, turmeric EO showed potent antibacterial activity against *Helicobacter pylori*, *Bacillus cereus*, *B. coagulans*, *B. subtilis*, *Staphylococcus aureus*, *Escherichia coli*, *Vibrio parahaemolyticus*, *Proteus mirabilis*, and *Pseudomonas aeruginosa* [189,190]. It also showed strong antifungal effects against *Aspergillus flavus*, *A. niger*, *A. parasiticum*, *Rhizoctonia solani*, *Helminthosporium oryzae*, *Trichoconis padwickii*, *Curvularia lunata*, *C. pallescens*, *C. trifolii*, *Fusarium verticillioides*, *F. moniliforme*, *F. oxysporum*, *Penicillium digitatum*, *Alternaria dianthi*, *Trichophyton longifusus*, and *Colletotrichum falcatum* [23,77,189,191,192]. In addition, *C. longa* EO was reported to have antiaflatoxigenic activities [76]. Turmeric EO exhibited insecticidal activity against the white termite (*Odontotermes obesus*) [37,193,194] as well as insect-repellent activities [194]. It showed repellency against both day- and night-biting mosquitoes [195]. Turmeric oil and *ar*-turmerone isolated from the oil displayed mosquitocidal activity against *Aedes aegypti* larvae (LD_100_ = 50 µg/mL) [194] and *Anopheles quadrimaculatus*. Moreover, turmeric EO inhibited the germination and growth of *Avena fatua* L., *Echinochloa crus-galli* (L.) Beauv, *Allium cepa* L., and *Phalaris minor* Retz [189]. Turmeric-leaf EO showed cytotoxic activity against breast-tumor (Hs578T) and prostate-tumor (PC-3) cells [94]. It also showed antibacterial, antifungal, antiaflatoxigenic, and mosquitocidal activities [89,94,194].

### 3.2. Zedoary (C. zedoaria) Essential Oil

*Curcuma zedoaria* EO showed potent radical-scavenging effects evaluated by DPPH assay [7,23,111,196,197]. The strong antioxidant activity of *C. zedoaria* EO is utilized in the food industry to minimize or prevent lipid oxidation. Zedoary EO also showed potent, selective cytotoxic activity and inhibited the proliferation of human cervical cancer (SiHa), colorectal cancer (SNU-1), human hepatoma (HepG2) [198], human gastric adenocarcinoma (AGS) [114], hepatic stellate cells [110], mouse melanoma (B16BL6) cells, human hepatoma (SMMC-7721) cells, and HL-60 cells [7,110]. It is worth noting that normal endothelial cells were less sensitive to zedoary EO than cancer cells in the in vitro assays [200]. The cytotoxic activity of zedoary EO is mediated by efficiently inhibiting monocytic differentiation, inhibiting cell proliferation, arresting cell cycle and inducing apoptosis [109,110,234]. The oil exhibited efficient cytotoxic effects against nonsmall cell lung carcinoma (NSCLC) cells via inducing apoptosis [199]. Zedoary EO showed antiproliferative activity against human colon-cancer cells (HCT116) by causing senescence and apoptosis in a dose- and time-dependent manner [235]. Zedoary EO in a combination with paclitaxel synergistically enhanced their antitumor activity and increased the apoptosis of human ovarian cancer (SKOV3) cells [202]. Zedoary EO (i.p.) significantly inhibited the growth of human lung-cancer cells (H1299) in vivo via inhibiting protein kinase B (Akt)/nuclear factor-kappa B (NF-κB) signaling pathways [199]. Zedoary EO was reported to inhibit angiogenesis in vitro and in vivo, which results in tumor inhibition [200]. Zedoary EO strongly inhibits vascular endothelial growth factor (VEGF)-induced angiogenesis in vitro and tumor angiogenesis in vivo via downregulating matrix metalloproteinases [200]. In rodent experiments, zedoary oil showed antitumor action in hepatoma-transplanted rats [203]. In addition, it has been used clinically in China for treating hepatic carcinoma [201]. In China, zedoary oil is used for treating gynecologic inflammation, monilial vaginitis, and tumors [236]. Zedoary EO is also known for its hypoglycemic effects [204]. In a study performed on streptozotocin-induced hyperglycemic Wistar rats, oral administration of the oil for seven days was able to significantly decrease blood-glucose levels and prevent gingivitis [204]. Zedoary EO has been used for oral-health maintenance because of its antimicrobial, hypoglycemic, and anti-inflammatory properties [14], which can help in reducing gingival inflammation. Zedoary EO exhibited antimicrobial activity against *Vibrio parahaemolyticus*, *Staphylococcus aureus*, *Bacillus cereus*, *Salmonella typhimurium*, and *Pseudomonas aeruginosa* [110]. It also demonstrated antifungal activity against *Colletotrichum falcatum* [37] and good insecticidal activity against the sugarcane pest, *Odontotermes obesus* Rhamb [37]. Zedoary oil displayed larvicidal effects against the malaria vector, *Anopheles dirus* (LC_50_ = 29.69 ppm), and the hemorrhagic fever vector, *Aedes aegypti* (LC_50_ = 31.87 ppm) [129].

### 3.3. Curcuma aeruginosa Essential Oil

*Curcuma aeruginosa* EO showed antiandrogenic [30], antinociceptive, antipyretic, and anti-inflammatory activities [15]. Topical application of *C. aeruginosa* extract (5% *w*/*w*) stimulated hair regrowth on patients with androgenic alopecia [205]. In a randomized controlled trial, *C. aeruginosa* rhizome extract promoted hair regrowth in bald males [205]. The bioactive compounds were identified as sesquiterpenes, with germacrone being the most potent [137]. Coapplication of *C. aeruginosa* EO, hexane extract, and germacrone improved the skin penetration of minoxidil, a hair-growth promoter approved as topical treatment of androgenic alopecia [30]. Skin penetration of minoxidil with EO, hexane extract, and germacrone was enhanced 20-fold, 4-fold, and 10-fold, respectively [30]. In a randomized, double-blinded trial, *C. aeruginosa* rhizome EO formulated as a lotion (1% and 5% *w*/*w* EO) was reported to safely and effectively slow the growth of axillary hair and to rapidly and robustly increase axillary skin brightness (within three weeks) [206]. Interestingly, these effects persisted for two weeks after ending the treatment. The rhizome EO of *C. aeruginosa* showed potent antibacterial activity against *Enterococcus faecalis* (MIC = 6.25 µg/mL) [29] and *Streptococcus mutans* (MIC = 15.63 µg/mL) and as a teeth-biofilm degradation [207], which makes it a good candidate as a natural antibacterial agent in a mouthwash or a toothpaste. It exhibited moderate antibacterial activity against *Staphylococcus aureus* (MIC = 125 µg/mL) and *Bacillus cereus* (MIC = 125 µg/mL) [2]. The oil showed antifungal activity against *Candida albicans* (MIC = 250 µg/mL) [2]. The oil showed weak inhibitory effect against *Mycobacterium tuberculosis* strain H37Ra (MIC = 2500 µg/mL) when tested by green fluorescent protein microplate assay [29]. The oil also showed strong radical-scavenging power evaluated by DPPH scavenging assay (EC_50_ = 24.32 µg/mL) due to the presence of germacrone and curzerenone [29].

### 3.4. Curcuma zanthorrhiza Essential Oil

*Curcuma zanthorrhiza* EO possesses antiproliferative [220], anti-inflammatory, antidiuretic, hypotensive, antihepatotoxic, antioxidant, antibacterial, and antifungal activities [141]. The anti-inflammatory activity of *C. zanthorrhiza* mainly depends on its germacrone content [221]. The oil effectively inhibited copper-mediated oxidation of LDL in thiobarbituric acid reactive substances assay (IC_50_ = 2.2 ± 0.1 µg/mL) [71]. The rhizome EO of *C. zanthorrhiza* showed antibacterial activity against *Staphylococcus aureus* (ZOI = 11.53 ± 0.27 mm) and antifungal activity against *Candida albicans* (ZOI = 7.29 ± 0.17 mm) [2]. Wicaksono et al. [222] reported analgesic effects (both central and peripheral) for *C. zanthorrhiza* EO, curcuminoid, and a combination of both in mice using the formalin test. Addition of *C. zanthorrhiza* EO (0.2%) or hexane-soluble fraction (0.5%) to rats' diet resulted in lower liver-triglyceride level and lower hepatic fatty-acid synthase activity [108]. *C. zanthorrhiza* hexane-soluble fraction also caused a decrease in food intake and an increase in relative liver weight in rats, while the oil did not [108]. *C. zanthorrhiza* had hypoglycemic activity and hypotriglyceridemic activity in diabetic rats [223,224], which was attributed to the activity of α-curcumene [108]. The hexane extract of *C. zanthorrhiza* exhibited antioxidant, larvicidal, cytotoxic, and antimicrobial activities [146].

### 3.5. Wild Turmeric (Curcuma aromatica) Essential Oil

Wild turmeric EO is reported to promote blood circulation, remove blood stasis, and treat cancers [148]. *C. aromatica* EO showed a remarkable anti-inflammatory activity via suppressing the production of proinflammatory cytokines including protein kinase C (PKC), Akt, tumor-necrosis factor-α (TNF-α), cyclooxygenase-2 (COX-2), NF-κB, and IκB kinase (IKK) in vivo in 12-*O*-tetradecanoylphorbol-13-acetate (TPA)-induced edema model [47,49]. It is thought that turmerone, *ar*-turmerone, 8,9-dehydro-9-formyl-cycloisolongifolene, *ar*-curcumene, α-zingiberene, and germacrone are responsible for the anti-inflammatory activity of *C. aromatica* EO [15]. The oil showed good cytotoxic activities against LNCaP HepG2, and B16 cell lines [47,49]. The oil can also suppress the growth of hepatoma cells in vivo and in vitro [214]. The oil was reported to induce apoptosis in NSCLC cells [208]. *ar*-Tumerone, turmerone, and curdione from *C. aromatica* EO have in vitro and in vivo antiproliferative effect on laryngeal cancer (Hep-2) cells [210]. Wild turmeric oil infused via hepatic artery inhibited hepatic tumors in patients with primary liver cancer [213], rats with transplanted hepatoma [211], and mice [212]. *C. aromatica* EO showed antiproliferative effects on hepatoma by inhibiting its growth in mice (51–52%) via decreasing the DNA synthesis of hepatocellular carcinoma and shrinking the nucleus area [212]. The antitumor activity of wild turmeric EO was attributed to the presence of β-elemene, curcumol, and curdione [237]. *C. aromatica* EO showed hepatic chemopreventive activity against hepatocellular carcinoma both in vivo and in vitro [214]. Pretreatment with *C. aromatica* oil (100 mg/kg for 3 days) protected mice from hepatic injury from inflammation and oxidative damage induced by concanavalin A, which can decrease the incidence of hepatocellular carcinoma.

Moreover, *C. aromatica* oil treatment (100 mg/kg, 200 mg/kg, 300 mg/kg body weight, i.p.) showed protective and antifibrosis activities in renal interstitial fibrosis rats in a time-dependent manner. Its mechanism involved inhibiting some metabolic pathways, including glycolysis, lipids metabolism, and methylamine metabolism [215]. *C. aromatica* EO showed potent radical-scavenging activities in the DPPH radical scavenging assay (IC_50_ = 1.57–21.36 µg/mL), 2,2’-azinodi (3-ethyl benz-thiazoline sulfonic acid) diammonium salt (ABTS) radical scavenging assay, and β-carotene bleaching tests in a concentration-dependent manner [47,50,54,147] due to the presence of 8,9-dehydro-9-formyl-cycloisolongifolene, germacrone [238], camphor, and borneol [217]. Because of its potent antioxidant activity, wild turmeric EO inhibited the development of esophageal cancer when administered intraperitoneally to rats [209]. In China, direct infusion of *C. aromatica* EO into the hepatic artery has been used in the clinical treatment of liver cancers [201]. Curdione from *C. aromatica* EO exhibited antiplatelet aggregation and antithrombotic activities both in vitro and in vivo in a concentration-dependent manner [216]. The oil also showed significant antibacterial activity against *Staphylococcus aureus*, *Listeria monocytogenes*, *Bacillus subtilis*, *Pseudomonas aeruginosa*, *Salmonella typhimurium*, and *Escherichia coli* [47,54,217], as well as antifungal activity against *Candida albicans* and *Saccharomyces cerevisiae* [47]. In pediatrics, the oil is used for treating acute upper-respiratory infections, viral myocarditis, and acute pneumonia [234]. *C. aromatica* EO also showed insecticidal effects against the booklouse *Liposcelis bostrychophila* Badonnel [56]. The rhizome volatile oil and hexane crude extract of *C. aromatica* showed larvicidal, adulticidal, and repellent activities against the hemorrhagic fever vector, *Aedes aegypti*, with the oil being more potent [52]. Hexane, dichloromethane, and methanol extracts of *C. aromatica* showed cardioprotective effects against isoproterenol-induced acute myocardial ischemia in rats [218]. Moreover, the extracts also showed antidiabetic activity via antiglycation and inhibiting α-amylase [51]. The leaf EO of *C. aromatica* showed antifungal activity against *Colletotrichum falcatum* and good insecticidal activity against the sugarcane pest, *Odontotermes obesus* Rhamb [37]

### 3.6. Curcuma phaeocaulis Essential Oil

*Curcuma phaeocaulis* EOs and extracts have been reported to possess strong antimicrobial and antifungal activities [219]. *C. phaeocaulis* EO showed moderate–strong antifungal activities against *Candida albicans* and *Saccharomyces cerevisiae*, and moderate–strong antibacterial activity against *Escherichia coli*, *Pseudomonas aeruginosa*, and *Staphylococcus aureus* [100]. These activities are thought to be due to the presence of germacrone, eremanthin, ar-curcumene, α-caryophyllene, and 8,9-dehydro-9-formyl-cycloisolongifolene [110]. The oil also showed strong radical-scavenging activities evaluated by DPPH assay (IC_50_ = 2.17–22.36 µg/mL) due to the high 8,9-dehydro-9-formyl-cycloisolongifolene, curzerene, 1,8-cineole, and germacrone content [100]. In addition, *C. phaeocaulis* EO exhibited a good anti-inflammatory activity through downregulating TNF-α and COX-2 expression in a TPA-induced skin-inflammation model [100]. Most the *C. phaeocaulis* oils tested from China showed strong cytotoxic activities against LNCaP and B16 cell lines (IC_50_ = 20.36–79.44 µg/mL) due to the presence of 8,9-dehydro-9-formyl-cycloisolongifolene, while some samples from a different region in China showed weak cytotoxic activity (IC_50_ = 245.19–245.30 µg/mL) [100].

### 3.7. Curcuma amada Essential Oil

Mango ginger possess central nervous system depressant, analgesic, antioxidant, anti-inflammatory, antiplatelet, cytotoxic, hypotriglyceridemic, antibacterial, and antifungal activities [157]. *C. amada* rhizome EO and ethanolic extracts showed hepatoprotective effects against carbon tetrachloride-induced hepatotoxicity in male Wister rats mainly due to their strong antioxidant activities [156]. The supercritical CO_2_ extract of mango ginger was selectively cytotoxic to human glioblastoma cell line (U-87MG; IC_50_ = 4.92 µg/mL). The extract was able to induce apoptosis in brain-tumor cells in a dose-dependent manner [226]. The supercritical CO_2_ extract also exhibited antitumor effects in human glioblastoma multiforme cells both in vitro and in nude mice xenografts. It was synergistic with irinotecan, a chemotherapy drug. In fact, treatment with a combination of irinotecan and *C. amada* extract showed almost a complete inhibition of tumor growth [227]. The extract was highly cytotoxic to human alveolar (SJRH30) and embryonal (RD) rhabdomyosarcoma cell lines, with IC_50_ values of 7.13 µg/mL and 7.50 µg/mL, respectively. It also showed synergistic cytotoxic effects with vinblastine and cyclophosphamide via inducing a higher percentage of apoptosis than individual agents [225]. *C. amada* EO showed strong antioxidant activity as evaluated by DPPH radical scavenging assay, total antioxidant assay, ferric-reducing antioxidant power and nitric oxide scavenging assay [229]. Moreover, *C. amada* EO showed 100% insect repellency and direct insecticidal effects against laboratory bred houseflies, *Musca domestica* L. [230]. The oil was antibacterial against *Staphylococcus aureus*, *Escherichia coli*, *Klebsiella pneumoniae*, *Pseudomonas aeruginosa*, *Salmonella paratyphi*, *Vibrio cholera*, *Enterobacter aerogenes*, *Streptococcus pneumoniae*, *Bacillus subtilis*, *Bacillus cereus*, *Proteus mirabilis*, *Proteus vulgaris*, and *Serratia marcescens* [229]. Organic extracts of mango ginger also demonstrated antibacterial effects against *E. coli*, *Bacillus subtilis*, *B. cereus*, *Staphylococcus aureus*, *Micrococcus luteus*, *Listeria monocytogenes*, *Enterococcus fecalis*, and *Salmonella typhi* [156]. *C. amada* EO showed antifungal activity against sugarcane pathogenic fungi such as *Physalospora tucumanensis*, *Sclerotium rolfsii*, *Helminthosporium sacchari*, and *Cephalosporium sacchari* [228].

### 3.8. Bioactivities of Other Curcuma Essential Oils

The EO of *C. mangga* showed strong antibacterial activities against *Staphylococcus aureus* (MIC = 1.2 µL/mL), *Bacillus cereus* (MIC = 11.1 µL/mL), *P. aeruginosa* (ZOI = 9.0 mm), and *E. coli* (ZOI = 7.0 mm), as well as antifungal activity against *Candida albicans* (MIC = 3.7 µL/mL) and *Cryptococcus neoformans* (MIC = 0.1 µL/mL) [28]. The rhizome EO of *C. glans* showed antibacterial activity against *Staphylococcus aureus* (ZOI = 17.24 ± 0.07 mm) and antifungal activity against *C. albicans* (ZOI = 7.27 ± 0.17 mm) [2]. *C. singularis* rhizome EO displayed moderate antibacterial activity against *Bacillus subtillis* (MIC = 100 µg/mL) and *E. coli* (MIC = 200 µg/mL) [106]. The rhizome and leaf EOs of *C. angustifolia* showed significant antioxidant activities with the leaf oil being more potent [45]. The root and rhizome EOs of *C. alismatifolia* showed strong DPPH radical scavenging activity (EC_50_ = 10.2 ± 0.94 µg/mL and 11.48 ± 1.02 µg/mL, respectively) and ferric-reducing power activity (EC_50_ = 0.12 ± 0.03 µg/mL) [36]. *C. elata* EO showed a potent DPPH radical-scavenging activity and was cytotoxic to LNCaP (IC_50_ = 18.4 μg/mL) and HepG2 (IC_50_ = 167.75 μg/mL) [49]. *C. sichuanensis* EO and *C. rubescens* EO showed potent DPPH radical-scavenging activities (IC_50_ = 4.52 μg/mL and 22.32 μg/mL, respectively) [49,50]. *C. sichuanensis* oils (68.43% inhibition), *C. nankunshanensis* oils (55.23% inhibition), and *C. elata* oils (54.64% inhibition) exhibited a good anti-inflammatory effects in TPA-induced edema model [49]. They inhibited the production of proinflammatory cytokines including PKC, Akt, TNF-α, COX-2, NF-κB, and IKK [49]. The EO from *C. kwangsiensis* possesses antitumor, antioxidant, anti-inflammatory, bactericidal, antifungal, and antiviral activities [62,63]. *C. kwangsiensis*, *C. yunnanensis*, *C. nankunshanensis*, *C. sichuanensis*, and *C. rubescens* EOs were cytotoxic to LNCaP (IC_50_ = 1.3–16.6 µg/mL), B16 (IC_50_ = 4.4–147.4 μg/mL), and HepG2 (IC_50_ = 153.1–198.2 µg/mL) [49,63]. *C. purpurascens* EO showed strong antiproliferative activity against human colorectal-cancer cells (HT-29; IC_50_ = 4.9 ± 0.4 μg/mL), and weak cytotoxicity against human lung-cancer (A549; IC_50_ = 46.3 ± 0.7 μg/mL), human cervical-cancer (Ca Ski; IC_50_ = 32.5 ± 1.1 μg/mL), and HCT116 cells (IC_50_ = 35.0 ± 0.3 μg/mL) [103].

## 4. Toxicity and Safety

In general, *Curcuma* EOs are nontoxic, nonmutagenic, noncarcinogenic and nonphototoxic [125,239]. Turmeric EO has been classified as generally recognized as safe (GRAS) [125]. Undiluted turmeric rhizome oil was slightly irritating to rabbits, but was not irritating to mice. When tested at 4% on 25 volunteers, it was neither irritating nor sensitizing [239]. There is a possible drug interaction when used orally, especially with antidiabetic medications [125]. The acute dermal LD_50_ of turmeric rhizome oil was >5 g/kg in rabbits, and the acute oral LD_50_ was >5 g/kg in rats [239]. When administered intraperitoneally (i.p.) at doses higher than 28 mg/kg/day, 20–36% of normal and streptococcal cell wall-injected animals died after two weeks of treatment, while lower vehicle or oil doses (≤2.8 mg/kg/day) caused no deaths [182]. Oral administration of a dose of turmeric oil that is 20-fold higher than the lowest effective i.p. doses was nontoxic [182]. No hazards or adverse skin reactions were reported for turmeric-leaf EO; however, the α-phellandrene chemotype might cause skin sensitization on oxidation.

Zedoary EO has GRAS status [125]. No acute toxicity or adverse reactions were reported for the zedoary oil; however, its consumption may interfere with gestation and may induce abortion [125]. For this reason, the oil and extracts are strictly prohibited during pregnancy and should be avoided during breastfeeding. Zedoary EO showed obvious embryotoxicity ex vivo and reproductive toxicity in animal and developmental experiments [109,200]. In addition, treatment with aqueous extracts of *C. zedoaria* rhizome (10 g/kg/day for 20 days) exhibited reproductive toxicity in pregnant mice [240]. Chinese zedoary EO prevented implantation in dose-dependent manner. When given i.p. (300 mg/kg) to female rats on gestational days 7–9, it prevented 77% of pregnancies, and when administered intravaginally to female rabbits, it prevented 16% and 100% of pregnancies at 60 or 400 mg/kg/day on gestational days 5–9 and 2–4, respectively [125]. It was suggested that the embryotoxic effect of zedoary EO might be caused by its sesquiterpenoids that can block VEGF-mediated angiogenesis [109]. However, no direct evidence was found to link any of the oil components to its antifertility effect. Decoctions and ethanol extracts of zedoary rhizomes also have antifertility effects [241].

No hazards, acute toxicity, or adverse reactions were reported for the wild turmeric (*C. aromatica*), the mango ginger (*C. amada*), and the pink-and-black curcuma (*C. aeruginosa)* rhizome oils [125,206]. No information found for the toxicity and safety of other *Curcuma* oils.

## 5. Bioactivity and Safety of Individual Key Components

A summary of the biological activities of key components of Curcuma essential oils is presented in Table 3.

*ar*-Turmerone, α-turmerone, and β-turmerone are major constituents of turmeric rhizome oil. *ar*-Turmerone displayed strong in vitro antiplatelet aggregation activity [174], antimutagenic [178], and potent hypoglycemic activity against α-glucosidase and α-amylase [167]. *ar*-Turmerone effectively inhibited copper-mediated oxidation of LDL (IC_50_ = 2.2 ± 0.1 µg/mL) [71]. It also showed neuroprotective effect through inhibiting microglia activation, increasing neural-stem-cells proliferation, and promoting neuronal differentiation [244]. *ar*-Turmerone, isolated from turmeric EO, showed potent cytotoxic activity against several cell lines including HL-60 [245], human leukemia (K-562), rat leukemia (RBL-2H3), and mouse leukemia (L-1210) [246], HeLa [220], HepG2, and human lymphoma (U937) [247] via inducing apoptosis and internucleosomal DNA fragmentation. It was effective against sarcoma 180 ascites (connective tissue cancer) in mice at a dose of 50 mg/kg [248]. *ar*-Turmerone is also a potent anti-inflammatory agent; it inhibits the production of inflammatory cytokines [242]. *ar*-Turmerone exhibited a potent inhibition of both inducible COX-2 (IC_50_ = 5.2 µg/mL) and iNOS (IC_50_ = 3.2 µg/mL) as part of its cancer chemopreventive action [249]. Turmerone-enriched turmeric oil protected from LPS-induced inflammation in human monocytes (THP-1), murine macrophages (J774.2), and Swiss mice [243]. Turmerone isolated from *C. longa* showed antivenom [188] and insect-repellent activities [120]. It also had a strong antibacterial activity against *Clostridium perfringens* [250], and a strong antifungal activity against *Aspergillus flavus* [251]. No acute toxicity was found for *ar*-tumerone, but it might be nontoxic, similar to turmeric rhizome oil. However, *ar*-turmerone has been classified as potential for allergic skin reaction (H317) and eye irritation (H319) [287].

Curdione, the main component in *C. aromatica*, *C. nankunshanensis*, and *C. trichosantha* EOs significantly suppressed the proliferation of human breast-cancer cells (MCF-7) via inducing cell apoptosis and impairing mitochondrial-membrane potential [252]. Curdione, from zedoary EO, inhibited PGE2 production in LPS-stimulated mouse macrophage RAW 264.7 cells (IC_50_ = 1.1 μM) through suppressing COX-2 expression [253]. Curdione is also known for its outstanding antibacterial and antifungal activities [72]. As far as we are aware, there are no known hazards associated with curdione.

1,8-Cineole possesses strong antioxidant [254,255] and anticarcinogenic [256] activities. The antioxidant activity of 1,8-cineole was associated with eliminating the 2,3,7,8-tetrachlorodibenzo- *p*-dioxin-induced oxidative stress in rats [288]. 1,8-Cineole is not a skin irritant, convulsant, or photosensitizing [125]. There is no evidence of carcinogenesis or teratogenesis in rodents. It is nonmutagenic, nongenotoxic, and nonfetotoxic in normal doses [125]. High oral doses of cineole are toxic, especially to children. 1,8-Cineole neurotoxicity resulting from nasal instillation is expressed primarily as irritated mucous membranes, tachycardia, dyspnea, nausea, vomiting, vertigo, muscular weakness, drowsiness, and coma [289]. The acute dermal LD_50_ of 1,8-Cineole was >5 g/kg in rabbits, while the acute oral LD_50_ was 2.48 g/kg in rats [290].

β-Caryophyllene is nontoxic, nonmutagenic and antitumor. It inhibited the growth of myelogenous leukemia cells (IC_50_ = 98.0mM; 20.4 μg/mL), HL-60 cells (IC_50_ = 19.31 μg/mL), human melanoma cells (IC_50_ = 20.10 μg/mL), and renal cell adenocarcinoma cells (IC_50_ = 21.81 μg/mL) [125,257,258,259]. It was moderately cytotoxic against human-breast and cervical cancer cell lines, and human and mouse melanoma cells [291]. Survival was considerably increased after 4 daily intraperitoneal doses of 20 mg/kg β-caryophyllene in mice with ascites tumors [292]. β-Caryophyllene showed antileishmanial activity against *L. amazonensis* amastigotes (IC_50_ = 1.3µg/mL) [260], and antitrypanosomal activity against *Trypanosoma cruzi* epimastigotes (IC_50_ = 78.4µM), trypomastigotes, and amastigotes (IC_50_ = 63.7 µM) [261]. β-Caryophyllene is a weak skin allergen, and its oxidation does not increase its allergenicity. Undiluted β-caryophyllene was irritating to rabbit skin while when tested at 4%, it was neither irritating nor sensitizing on 25 volunteers [239]. β-Caryophyllene induced allergic responses in 10 (0.6%) of 1,606 consecutive dermatitis patients when tested at 5% [293]. When tested at 3%, oxidized β-caryophyllene (about 25% β-caryophyllene and 75% caryophyllene oxide) showed positive reaction in 8 (0.5%) of 1,511 consecutive dermatitis patients, one positive reaction in 21 dermatitis patients hypersensitive to fragrance materials, and none in 66 hand-eczema patients [294]. β-Caryophyllene was not mutagenic in *Salmonella typhimurium* strains TA98 and TA100, and was antimutagenic in several assays [295]. The acute oral LD_50_ of β-caryophyllene was >5 g/kg in rats, and the acute dermal LD_50_ was >5 g/kg in rabbits [239].

β-Myrcene possesses strong antimutagenic [262], chemopreventive [263], antiproliferative [264,265], and antioxidant [266] effects. It is nonirritant, nonallergenic, nontoxic, and nongenotoxic [125]. Undiluted β-myrcene was moderately irritating to rabbits, but was neither irritating nor sensitizing to 25 volunteers when tested at 4% [239]. Oxidized myrcene (tested at 3% and containing 30% myrcene) showed reaction in only 0.07% in a multicenter study involving 1,511 consecutive dermatitis patients [294]. The acute oral LD_50_ of β-myrcene was >5 g/kg in rats, and the acute dermal LD_50_ was >5 g/kg in rabbits [239]. The oral “no observed adverse effect level” (NOAEL) of myrcene in rats was 300 mg/kg [296]. Rodent studies suggest that β-myrcene might carry a risk of carcinogenesis. When administered by gavage, β-myrcene increased the occurrences of hepatocellular carcinoma and hepatoblastoma in male mice, incidences of hepatocellular adenoma or carcinoma in female mice, and incidences of renal tubule adenoma or carcinoma in male rats, and induced rare renal tubule adenomas in female rats [297,298]. β-Myrcene is not genotoxic. As a component in essential oils used in aromatherapy, β-myrcene does not represent a level of fetotoxicity that would cause any problem. β-Myrcene may cause skin (H315) or eye irritation (H319), however [299].

Germacrone showed anti-inflammatory [131,267], antiandrogenic [137], and antimicrobial [28] activities. Germacrone from *C. aeruginosa* has been shown to increase skin penetration of minoxidil [30]. Germacrone exhibited antiproliferative activity against human breast-cancer cell lines (MCF-7 and MDA-MB-231) in a dose-dependent manner [268], as well as human glioblastoma cell lines (U-87 and U-251) [269] and human hepatoma cells via inducing cell-cycle arrest and apoptosis [270]. Germacrone from *C. aromatica* EO possessed antitumor effects through a similar mechanism [270]. Germacrone from zedoary EO exhibited strong antioxidant activity and was able to relieve the oxidative stress induced by hydrogen peroxide in mouse neuroblastoma (NG108-15) cells [271]. It inhibited the carrageenin-induced edema in rats, as well as acetic acid-induced vascular permeability and writhing symptoms in mice [221]. Additionally, germacrone effectively inhibited the growth of *Pseudomonas aeruginosa* (MIC = 15.6μg/mL) [272]. Germacrone may cause skin (H315) or eye irritation (H319) [300].

Xanthorrhizol has antioxidant, anti-inflammatory, antitumoral, hepatoprotective, neuroprotective, nephroprotective, estrogenic, and antibacterial properties [273,274]. Pretreatment with xanthorrhizol (p.o., 200 mg/kg/day for 4 days), significantly reduced the cisplatin-induced nephrotoxicity in mice [273]. Xanthorrhizol showed cancer chemopreventive action via potently inhibiting both COX-2 (IC_50_ = 0.2 µg/mL) and iNOS (IC_50_ = 1.0 µg/mL) [249]. Xanthorrhizol was antiproliferative to MCF-7 (EC_50_ = 1.71 µg/mL) and HepG2 cells (IC_50_ = 4.17 µg/mL) through inducing apoptosis [274]. Xanthorrhizol (at 50 mg/kg) was active against sarcoma 180 ascites in mice [248]. Intraperitoneal administration of xanthorrhizol (0.1, 0.2, 0.5, and 1.0 mg/kg for 2 weeks) inhibited the formation of lung-tumor nodules in mice by 36%, 63%, 61%, and 52%, respectively [275]. Xanthorrhizol strongly inhibited copper-mediated oxidation of human LDL (IC_50_= 0.4 ± 0.1 µg/mL) [71]. Although the toxicological properties have not been thoroughly investigated, xanthorrhizol may cause skin or eye irritation and may damage fertility or the unborn fetus (H360) [301].

β-Elemene inhibited the proliferation of several cancer cell lines [302]. It was cytotoxic to HL-60 cells (IC_50_= 27.5 µg/mL), K-562 (IC_50_ = 81 µg/mL) cells, peripheral blood leukocytes (IC_50_ = 254.3 µg/mL) [237], and human laryngeal-cancer cells in vitro and in vivo [210] in a dose-dependent manner via inducing apoptosis [303]. β-Elemene selectively inhibited the growth of human non-small-cell lung-cancer cells and human ovarian-cancer cells [302]. Moreover, it was able to overcome the cisplatin-resistance developed in cancer cells [302]. β-Elemene showed strong antiangionenic effects. At doses of 20 and 50 mg/kg/day for 21 days, it suppressed VEGF expression in B16F10 melanoma cells in mice, and repressed VEGF-dependent tumor angiogenesis [276]. When used in vitro at 20 and 50 mM, β-elemene inhibited the VEGF-induced sprouting of rat aortic-ring vessels in chick embryo chorioallantoic membranes [276]. β-Elemene protected against carbon tetrachloride-induced liver fibrosis in rats through downregulating the expression of plasma endotoxin, serum TNF-α, and hepatic cluster of differentiation 14 (CD14) [277]. β-Elemene (i.p., 50 and 100 mg/kg) reduced angiogenesis in gastric-cancer stem-like cells [304]. In vivo experiments showed that β-elemene treatment suppressed the growth of brain, lung, breast, colon, cervix, and prostate cancers (IC_50_ = 47–95 µg/mL) [278]. In a clinical trial, β-elemene was effective in managing malignant pleural and peritoneal effusions with local pain, fever, and gastrointestional disturbance as the major adverse effects [237]. In another clinical trial that included 40 brain-cancer cases, β-elemene treatment reduced average tumor size by 61%, and four cases completely recovered [279]. No toxicity or dermal data were found for β-elemene; however, its antiangiogenic action might suggest caution in pregnancy.

Terpinolene showed potent DPPH-scavenging activity [280] and remarkable protection against LDL oxidation [125]. Terpinolene was chemoprotective against the in vitro formation of the carcinogen *N*-nitrosodimethylamine (NDMA) by 79% inhibition [263]. It was neither irritating nor sensitizing when tested at 20% on volunteers [305]. Terpinolene was the reason behind several cases of tea tree oil allergenicity [125]. Terpinolene was sensitizing to all of 16 dermatitis patients sensitive to tea tree oil when tested at 10% [306]. The acute oral LD_50_ of terpinolene was 4.4 mL/kg in rats and mice [305]. The skin-sensitization thresholds of terpinolene are not known, but the limited data available suggests minimal toxicity.

8,9-Dehydro-9-formyl-cycloisolongifolene showed a good DPPH radical-scavenging activity [281]. It was reported to inhibit Akt/NF-κB signaling pathways in H1299 cells [199]. Curcumol induced apoptosis in human lung adenocarcinoma ASTC-a-1 cells [282]. Curzerene showed excellent antioxidant [168] and anticancer activities [283]. β-Sesquiphellandrene demonstrated remarkable DPPH-scavenging activity [168]. It showed anticancer potential when compared with curcumin [284] and was cytotoxic to the mouse lymphocytic leukemia (L1210) cell line [307]. *ar*-Curcumene appears to be responsible for the antitumor effects of *C. zanthorrhiza* [248]. α-Phellandrene possesses antioxidant, antinociceptive, and anti-inflammatory effects [285,286].

## Figures and Tables

**Table 1:** Major volatile components (>5%) in different *Curcuma* spp.

Curcuma Species	Origin	Part Used (Extraction Method)	Major Components (>5%)	Reference
*C. aeruginosa* Roxb.	Pahang, Malaysia	Rhizome (SD)	8,9-Dehydro-9-formyl-cycloisolongifolene (35.3%), dihydrocostunolide (22.5%), velleral (10.0%), and germacrone (6.5%)	[28]
*C. aeruginosa* Roxb.	Ratchaburi, Thailand	Fresh rhizome (HD)	Germacrone (23.5%), curzerenone (11.8%) and 1,8-cineole (10.9%)	[29]
*C. aeruginosa* Roxb.	Phetchabun, Thailand	Powdered rhizome (HD)	1,8-Cineole (22.7%), germacrone (17.7%), furanodiene (11.4%), and β-pinene (8.0%)	[30]
*C. aeruginosa* Roxb.	Malaysia	Rhizome (HD)	1,8-Cineole (23.2%) and curzerenone (28.4%)	[31]
*C. aeruginosa* Roxb.	Malaysia	Rhizome (HD)	Curzerenone (24.6%), 1,8-cineole (11.0%), camphor (10.6%), zedoarol (6.3%), isocurcumenol (5.8%), curcumenol (5.6%), and furanogermenone (5.5%)	[32]
*C. aeruginosa* Roxb.	Chiang Mai, Thailand	Rhizome (HD)	Camphor (29.4%), germacrone (21.2%), borneol (7.3%), and germacrene B (5.2%)	[2]
*C. aeruginosa* Roxb.	Kerala, India	Rhizome (HD)	Curcumenol (38.7%) and β-pinene (27.5%)	[17]
*C. aeruginosa* Roxb.	Pahang, Malaysia	Rhizome (SE, MTBE)	Methenolone (16.6%), 8,9-dehydro-9-formyl-cycloisolongifolene (15.9%), labd-13-en-15-oic acid,8,12-epoxy-12-hydroxy-γ-lactone (10.8%), propiolic acid, 3-(1-hydroxy)-2 isopropyl-1,5-methylcyclohexyl) (7.8%), and 4-oxo-β-isodamascol (5.2%)	[33]
*C. aeruginosa* Roxb.	Phetchabun, Thailand	Rhizome (SE, hexane)	Dehydrocurdione (27.6%), curcumenol (15.1%), germacrone (10.2%), and gajutsulactone A (6.3%)	[30]
*C. aeruginosa* Roxb.	South India	Leaf (HD)	1,8-Cineole (17.7%), curzerenone (10.5%), furanogermenone (7.8%), camphor (7.5%), (*Z*)-3-hexenol (5.8%), and furanodienone (5.1%)	[34]
*C. aeruginosa* Roxb.	Vietnam	Leaf (HD)	Curzerene (16.2%), germacrone (13.6%), 1,8-cineole (13.5%), and camphor (5.7%)	[35]
*C. albiflora* Thwaites	Ratnapura, Sri Lanka	Rhizome (HD)	α-Pinene (14.5%), caryophyllene oxide (9.4%), and alconfor (5.1%)	[13]
*C. alismatifolia* Gagnep.	Prachin Buri, Thailand	Fresh root (HD)	(–)-Xanthorrhizol (52.4%) and *ar*-curcumene (27.4%)	[36]
	Prachin Buri, Thailand	Fresh rhizome (HD)	β-Curcumene (42.0%), (-)-xanthorrhixol (36.6%), and *ar*-curcumene (7.5%)	[36]
*C. amada* Roxb.	Andhra Pradesh, India	Rhizome (HD)	Myrcene (80.5%)	[37]
*C. amada* Roxb.	Uttarakhand, India	Rhizome (HD)	Myrcene (88.8%)	[38]
*C. amada* Roxb.	Northeastern India	Fresh rhizome (HD)	Myrcene (88.6%)	[39]
*C. amada* Roxb.	New Delhi, India	Rhizome (SD)	(*Z*)-β-Farnesene (21.9%), guaia-6,9-diene (19.8%), α-longipinene (14.8%), α-guaiene (14.5%), and camphor (5.5%).	[40]
*C. amada* Roxb.	Mysore, India	Fresh rhizome (HD)	(*E*)-Hydroocimene (15.9%), (*Z*)-hydroocimene (14.2%), myrcene (14.9%), and linalool (13.4%)	[41]
*C. amada* Roxb.	Lucknow, India	Rhizome (HD)	*ar*-Curcumene (28.1%), β-curcumene (11.2%), camphor (11.2%), curzerenone (7.1%), and 1,8-cineole (6.0%)	[42]
*C. amada* Roxb.	Uttarakhand, India	Leaf (HD)	Camphor (17.9%), *epi*-curzerenone (10.8%), curzerenone (9.5%), and isoborneol (7.3%)	[38]
*C. angustifolia* Roxb.	Central India	Rhizome (HD)	Xanthorrhizol isomer (12.7%), methyleugenol (10.5%), and palmitic acid (5.2%)	[43]
*C. angustifolia* Roxb.	Southern India	Rhizome (HD)	Germacrone (12.8%), camphor (12.3%), isoborneol (8.7%), and curdione (8.4%)	[43]
*C. angustifolia* Roxb.	Chiang Mai, Thailand	Root (HD)	β-Elemenone (65.0%)	[44]
*C. angustifolia* Roxb.	Chiang Mai, Thailand	Rhizome (HD)	Camphor (36.9%) and germacrone (31.5%)	[44]
*C. angustifolia* Roxb.	India	Rhizome (HD)	Curzerenone (72.6%)	[45]
*C. angustifolia* Roxb.	India	Leaf (HD)	Curzerenone (33.2%), 14-hydroxy-δ-cadinene (18.6%), and γ-eudesmol acetate (7.3%)	[45]
*C. aromatica* Salisb.	Northeast India	Rhizome (HD)	Camphor (32.3%), curzerenone (11.0%), α-turmerone (6.7%), *ar*-turmerone (6.3%), and 1,8-cineole (5.5%)	[46]
*C. aromatica* Salisb.	China	Rhizome (SD)	8,9-Dehydro-9-formyl-cycloisolongifolene (2.7–36.8%), germacrone (4.3–16.5%), *ar*-turmerone (2.5–17.7%), turmerone (2.6–18.4%), ermanthin (0.8–13.3%), β-sesquiphellandrene (0.3–11.3%), and *ar*-curcumene (0.3–10.5%).	[47]
*C. aromatica* Salisb.	Assam, India	Rhizome (SD)	Camphor (25.6%), curzerenone (10.9%), germacrone (10.6%), 1,8-cineole (9.3%), isoborneol (8.2%), and camphene (7.4%)	[48]
*C. aromatica* Salisb.	Kerala, India	Rhizome (HD)	Camphor (18.8%), camphene (10.2%), 1,8-cineole (10.1%), borneol (8.2%), and β-elemene (7.5%)	[17]
*C. aromatica* Salisb.	Yulin, China	Fresh rhizome (SD)	Curdione (50.6%) and germacrone (9.5%)	[49]
*C. aromatica* Salisb.	Japan	Dry rhizome (SD)	Curcumol (35.8%), 1,8-cineole (12.2%), *ar*-turmerone (7.0%), linalool (6.4%), humulene oxide (6.1%), and caryophyllene oxide (5.9%)	[50]
*C. aromatica* Salisb.	Kerala, India	Rhizome (HD)	Xanthorrhizol (26.3%), *ar*-curcumene (19.5%), and di-*epi*-α-cedrene (16.5%)	[51]
*C. aromatica* Salisb.	Ratnapura, Sri Lanka	Rhizome (HD)	Camphor (32.3%), curzerenone (11.0%), α-turmerone (6.7%), *ar*-turmerone (6.3%), and 1,8-cineole (5.5%)	[13]
*C. aromatica* Salisb.	Thailand	Rhizome (HD)	1*H*-3a,7-methanoazulene (30.0%), curcumene (25.7%), and xanthorrhizol (13.7%)	[52]
*C. aromatica* Salisb.	Thailand	Rhizome (SE, hexane)	Xanthorrhizol (35.1%), 1*H*-3a,7-methanoazulene (21.8%), and curcumene (13.8%)	[52]
*C. aromatica* Salisb.	Hebei, China	Dry root (HSME)	β-Elemene (6.3%), germacrone (5.6%), and arzingiberone (5.3%)	[53]
*C. aromatica* Salisb.	Hebei, China	Dry root (SD)	Germacrone (9.1%), curcumenol (8.5%), isocurcumenol (7.5%), and arzingiberone (5.1%)	[53]
*C. aromatica* Salisb.	Hebei, China	Dry root (SPME)	Curcumenol (8.9%), isocurcumenol (8.7%), germacrone (6.7%), 1-methoxy-4-(1-propenyl)-benzene (5.7%), and curzerenone (5.3%)	[53]
*C. aromatica* Salisb.	Assam, India	Leaf (SD)	1,8-Cineole (20.0%), camphor (18.0%) germacrone (11.8%), camphene (9.4%), limonene (8.6%), and isoborneol (6.4%)	[48]
*C. aromatica* Salisb.	Gorakhpur, India	Leaf (HD)	*p*-Cymene (25.2%), 1,8-cineole (24.0%), α-terpineol (8.1%), and 2-oxabicyclo (3,2,1) octane-1-,4-dimethyl-8-methylene (8.1%)	[37]
*C. aromatica* Salisb.	Northeast India	Leaf (HD)	Camphor (28.5%), curzerenone (6.2%), and 1,8-cineole (6.1%)	[46]
*C. aromatica* Salisb.	Kushtia, Bangladesh	Leaf (HD)	Camphor (26.3%), borneol (16.5%), vinyldimethylcarbinol (12.2%), caryophyllene oxide (6.3%), cubenol (5.6%), and cucumber alcohol (5.2%)	[54]
*C. aromatica* Salisb.	Assam, India	Petiole (SD)	Camphor (16.8%), 1,8-cineole (8.8%), caryophyllene oxide (8.7%), patchouli alcohol (8.4%), isoborneol (6.8%), and elsholtzia ketone (6.0%)	[48]
*C. aurantiaca* Zijp	Kerala, India	Fresh rhizome (HD)	Piperitenone (65.2%), 1,8-cineole (13.1%), and camphor (5.7%)	[55]
*C. aurantiaca* Zijp	Zhejiang, India	Fresh rhizome (HD)	1,8-cineole (15.3%), camphor (10.1%), germacrone (6.9%), β-elemene (6.3%), curzerene (6.7%), and β-elemenone (5.2%)	[56]
*C. caesia* Roxb.	Kerala, India	Rhizome (HD)	1,8-Cineole (30.1%), camphor (15.2%), *ar*-curcumene (14.8%), and camphene (8.2%)	[17]
*C. caesia* Roxb.	Central India	Rhizome (HD)	Camphor (28.3%), *ar*-turmerone (12.3%), (*Z*)-β-ocimene (8.2%), *ar*-curcumene (6.8%), and 1,8-cineole (5.3%)	[57]
*C. caesia* Roxb.	India	Leaf (HD)	1,8-Cineole (27.0%) and camphor (16.8%)	[58]
*C. elata* Roxb.	Guangzhou, China	Fresh rhizome (SD)	8,9-Dehydro-9-formyl-cycloisolongifolene (52.2%) and germacrone (14.0%)	[49]
*C. glans* K. Larsen and Mood	Chiang Mai, Thailand	Rhizome (HD)	Germacrone (15.8%), β-pinene (10.0%), camphor (10.0%), and 2-nonanol (6.9%)	[2]
*C. haritha* Mangaly and M. Sabu	Southern India	Rhizome (HD)	Camphor (36.0%), 1,8-cineole (13.9%), isoborneol (10.6%), curdione (6.9%), and camphene (5.7%)	[59]
*C. harmandii* Gagnep.	Vietnam	Rhizome (SD)	1,8-Cineole (4.5-12.5%), germacrone (9.0–20.5%), β-pinene (1.2–22.6%), β-elemene (6.5–11.3%), and isocurcumenol (3.7–13.4%)	[60]
*C. harmandii* Gagnep.	Vietnam	Root (SD)	Germacrone (24.4%), isocurcumenol (12.9%), and curcumenol (10.8%)	[60]
*C. harmandii* Gagnep.	Vietnam	Leaf (SD)	1,8-Cineole (13.5%), germacrone (11.5%), and curdione (36.8%)	[60]
*C. harmandii* Gagnep.	Vietnam	Stem (SD)	1,8-Cineole (21.8%), germacrone (15.5%), and curdione (25.3%)	[60]
*C. harmandii* Gagnep.	Vietnam	Flower (SD)	Curdione (27.0%) and an unidentified oxygenated sesquiterpene (12.3%)	[60]
*C. inodora* Blatt.	Malaysia	Fresh rhizome (HD)	Curzerenone (20.8%), germacrone (11.1%), curdione (7.5%), and 1,8-cineole (5.3%)	[61]
*C. inodora* Blatt.	Malaysia	Leaf (HD)	Curzerenone (16.9%), germacrone (7.5%), 1,8-cineole (5.3%), and farnesol (5.0%)	[61]
*C. kwangsiensis* S.G. Lee and C.F. Liang	Guangzhou, China	Fresh rhizome (SD)	α-Elemene (12.8%), germacrene D (8.2%), spathulenol (5.8%), curdinone (5.9%), and β-bisabolene (5.4%)	[49]
*C. kwangsiensis* S.G. Lee and C.F. Liang	Guangxi, China	Rhizome (HD)	Germacrone (13.2%), β-elemenone (12.8%), β-elemene (4.5–6.8%), curzerenone (5.6–7.6%), and curdione (3.0–6.0%)	[62]
*C. kwangsiensis* S.G.Lee and C.F.Liang	China	Rhizome (HD)	8,9-Dehydro-9-formyl-cycloisolongifolene (2.37–42.59%), germacrone (6.53–22.20%), and l-camphor (0.19–6.12%).	[63]
*C. longa* L.	Tamil Nadu, India	Dry rhizome (HD)	*ar*-Turmerone (53.1%), β-turmerone (6.4%), and α-turmerone (6.2%)	[64]
*C. longa* L.	Mumbai, India	Dry rhizome (HD)	*ar*-Turmerone + turmerone (68–70%) and curlone (12–15%)	[65]
*C. longa* L.	Kanpur, India	Fresh rhizome (HD)	*ar*-Turmerone (31.7%), α-turmerone (12.9%), β-turmerone (12.0%), and (*Z*)-β-ocimene (5.5%)	[66]
*C. longa* L.	Gorakhpur, India	Rhizome (HD)	*ar*-Turmerone (51.7%), β-bisabolene (10.7%), α-turmerone (11.9%), zingiberene (10.2%), and β-caryophyllene (5.6%)	[37]
*C. longa* L.	Gorakhpur, India	Fresh rhizome (HD)	*ar*-Turmerone (24.4%), α-turmerone (20.5%), and β-turmerone (11.1%)	[23]
*C. longa* L.	Gorakhpur, India	Dry rhizome (HD)	*ar*-Turmerone (21.4%), α-santalene (7.2%), *ar*-curcumene (6.6%), and santalenone (5.6%)	[23]
*C. longa* L.	Gorakhpur, India	Fresh rhizome (SE, ethanol)	α-Turmerone (53.4%), β-turmerone (18.1%), and *ar*-turmerone (6.2%)	[23]
*C. longa* L.	Gorakhpur, India	Dry rhizome (SE, ethanol)	*ar*-Turmerone (9.6%), α-santalene (7.8%), β-sesquiphellandrene (6.9%), α-turmerone (6.5%), and α-zingiberene (6.1%)	[23]
*C. longa* L.	Karnataka, India	Fresh rhizome (HD)	α-Turmerone (33.5%), *ar*-turmerone (21.0%), and β-turmerone (18.9%)	[67]
*C. longa* L.	Karnataka, India	Dry rhizome (HD)	*ar*-Turmerone (30.3%), α-turmerone (26.5%), and β-turmerone (19.1%)	[67]
*C. longa* L.	Karnataka, India	Cured rhizome (HD)	*ar*-Turmerone (28.3%), α-turmerone (24.8%), and β-turmerone (21.1%)	[67]
*C. longa* L.	Mysore, India	Rhizome (SE, hexane)	*ar*-Turmerone (21.4%), zingiberene (15.0%), (*Z*)-β-farnesene (14.0%), *ar*-curcumene (10.3%), turmerone (6.2%), and curlone (5.1%)	[22]
*C. longa* L.	Bangalore, India	Rhizome (HD)	Turmerone (44.1%), β-turmerone (18.5%), and *ar*-turmerone (5.4%)	[68]
*C. longa* L.	Gorakhpur, India	Dried rhizome (HD)	*ar*-Turmerone (49.1%) and α-turmerone (11.6%)	[69]
*C. longa* L.	Calicut, India	Rhizome (HD)	*ar*-Turmerone (31.1%), curlone (10.6%), turmerone (10.0%), and *ar*-curcumene (6.3%)	[70]
*C. longa* L.	Calicut, India	Root (HD)	*ar*-Turmerone (46.8%) and *ar*-curcumene (7.0%)	[70]
*C. longa* L.	Kuala Selangor, Malaysia	Fresh rhizome (HD)	*ar*-Turmerone (45.8%) and curcumenol (18.2%)	[71]
*C. longa* L.	Faisalabad, Pakistan	rhizome (SD)	*ar*-Turmerone (25.3 %), α-tumerone (18.3 %), and curlone (12.5 %)	[72]
*C. longa* L.	Pakistan	Rhizome (HD)	*ar*-Turmerone (38.6%), a-turmerone (8.9%), and β-turmerone (12.9%)	[73]
*C. longa* L.	Sichuan, China	Dried rhizomes (SD)	*ar*-Turmerone (49.0%), humulene oxide (16.6%), β-selinene (10.2%), and caryophyllene oxide (5.6%)	[50]
*C. longa* L.	China	Fresh rhizome (HD)	*ar*-Turmerone (0.9–42.9%), β-turmerone (5.1–42.5%), α-zingiberene (0.3–25.1%), *ar*-curcumene (1.2–15.7%), and β-sesquiphellandrene (0.1–14.9%)	[74]
*C. longa* L.	Sichuan, China	Rhizome (SFE)	α-Turmerone (40.8%), zingiberene (16.9%), β-turmerone (14.1%), *ar*-turmerone (11.0%), and β-sesquiphellandrene (10.0%)	[75]
*C. longa* L.	Mara Rosa, Brazil	Rhizome (HD)	*ar*-Turmerone (33.2%), α-turmerone (23.5%), and β-turmerone (22.7%)	[76]
*C. longa* L.	Mara Rosa, Brazil	Fresh rhizome (HD)	α-Turmerone (42.6%), β-turmerone (16%), *ar*-turmerone (12.9%), and α-phellandrene (6.5%)	[77]
*C. longa* L.	Minas Gerais, Brazil	Rhizome (SE)	(*Z*)-γ-Atlantone (33.4%), *ar*-turmerone (21.8 %), and (*E*)-γ-atlantone (18.7%)	[78]
*C. longa* L.	Minas Gerais, Brazil	Rhizome (HD)	(*Z*)-γ-Atlantone (44.0%), (*E*)-γ-atlantone (18.3%), and *ar*-turmerone (18.0%)	[78]
*C. longa* L.	Isfahan, Iran	Dry rhizome (HD)	*ar*-Turmerone (68.9%) and α-turmerone (20.9%)	[79]
*C. longa* L.	Brazil	Rhizome (SFE)	*ar*-Turmerone (51.9%) and (*E*)-γ-atlantone (19.6%)	[80]
*C. longa* L.	Brazil	Rhizome (HD)	*ar*-Turmerone (49.3%) and (*E*)-γ-atlantone (19.2%)	[80]
*C. longa* L.	Ondo, Nigeria	Fresh rhizome (HD)	Turmerone (35.9%), α-phyllandrene (15.5%), curlone (12.9%), 1,8-cineole (10.3%), and *ar*-turmerone (10.0%)	[81]
*C. longa* L.	Cameroon	Rhizome (HD)	α-Turmerone (43.1%), *ar*-turmerone (17.6%), and curlone (17.5%)	[82]
*C. longa* L.	Bhutan	Rhizome (HD)	α-Turmerone (30.0–32.0%), *ar*-turmerone (17.0–26.0%), and β-turmerone (15.0–18.4%)	[83]
*C. longa* L.	Reunion, France	Rhizome (SD)	α-Turmerone (21.4%), terpinolene (15.8%), zingiberene (11.8%), β-sesquiphellandrene (8.8%), *ar*-turmerone (7.7%), β-turmerone (7.1%), and β-caryophyllene (5.7%)	[84]
*C. longa* L.	North Central Nigeria	Fresh rhizome (HD)	β-Bisabolene (13.9%), (*E*)-β-ocimene (9.8%), myrcene (7.6%), 1,8-cineole (6.9%), α-thujene (6.7%), α-phellandrene (6.4%), limonene (5.3%), zingiberene (5.2%), and β-sesquiphellandrene (5.2%)	[85]
*C. longa* L.	North Indian Plains	Rhizome (HD)	1,8-Cineole (11.2%), α-turmerone (11.1%), β-caryophyllene (9.8%), *ar*-turmerone (7.3%), and β-sesquiphellandrene (7.1%)	[86]
*C. longa* L.	Kerala, India	Rhizome (HD)	1,8-Cineole (28.2%), β-elemene (8.2%), camphor (6.9%), α-farnesene (6.3%), and (*Z*,*Z*)-farnesol (5.2%)	[17]
*C. longa* L.	São Tomé and Principe	Rhizome (HD)	α-Phellandrene (15.5–30.4%), α-turmerone (12.2–23.9%), 1,8-cineole (10.2–23.0%), *ar*-turmerone (4.0–12.8%), β-turmerone (4.3–11.5%), and *p*-cymene (2.5–5.5%)	[87]
*C. longa* L.	Colombo, Sri Lanka	Rhizome (HD)	α-Phellandrene (18.2%), 1,8-cineole (14.6%), *p*-cymene (13.3%), and terpinolene (11.6%)	[13]
*C. longa* L.	Malaysia	Rhizome (HD)	Furanogermenone (53.1%), germacrone (9.6%) and β-elemene (8.8%), camphor (6.3%), and isofuranodiene (5.6%)	[88]
*C. longa* L.	Malaysia	Rhizome (HD)	α-Tumerone (45.3%), linalool (14.9%), and β-tumerone (13.5%)	[31]
*C. longa* L.	Calicut, India	Flower (HD)	*p*-Cymen-8-ol (26.0%) and terpinolene (7.4%)	[70]
*C. longa* L.	Reunion, France	Flower (SD)	Terpinolene (67.4%)	[84]
*C. longa* L.	Reunion, France	Leaf (SD)	Terpinolene (76.8%)	[84]
*C. longa* L.	Kanpur, India	Fresh leaf (HD)	α-Phellandrene (9.1%), terpinolene (8.8%), 1,8-cinceole (7.3%), undecanol (7.1), and *p*-cymene (5.5%)	[66]
*C. longa* L.	Kerala, India	Leaf (HD)	α-Phellandrene (24.4%), terpinolene (13.1%), *p*-cymene (11.1%), and 1,8-cineole (7.0%)	[89]
*C. longa* L.	Uttar Pradesh, India	Leaf (HD)	*p*-Cymene (25.4%), 1,8-cineole (18.0%), *cis*-sabinol (7.4%), and α-pinene (6.3%)	[90]
*C. longa* L.	Bangalore, India	Leaf (HD)	α-Phellandrene (53.4%), terpinolene (11.5%), and 1,8-cineole (10.5%)	[68]
*C. longa* L.	Calicut, India	Leaf (HD)	α-Phellandrene (32.6%), terpinolene (26%), 1,8-cineole (6.5%), and *p*-cymene (5.9%)	[70]
*C. longa* L.	Bhutan	Leaf (HD)	α-Phellandrene (18.2%), 1,8-cineole (14.6%), *p*-cymene (13.3%), terpinolene (11.6%), and β-pinene (7.2%),	[83]
*C. longa* L.	Nigeria	Leaf (HD)	α-Phellandrene (47.7%) and terpinolene (28.9%)	[91]
*C. longa* L.	Kerala, India	Leaf (HD)	β-Sesquiphellandrene (22.8%) and terpinolene (9.5%)	[92]
*C. longa* L.	Nainital, India	Leaf (SD)	Terpinolene (71.2%) and 1,8-cineole (6.2%)	[93]
*C. longa* L.	Southern Nigeria	Leaf (HD)	*ar*-Turmerone (63.4%), α-turmerone (13.7%), and β-turmerone (12.6%)	[94]
*C. longa* L.	Selangor, Malaysia	Leaf (PLE)	α-Phellandrene (13.8–20.7%), 1,8-cineole (14.4–15.1%), terpinolene (7.7–9.4%), and *p*-cymene (5.0–6.4%)	[95]
*C. longa* L.	Belem, Brazil	Fresh leaf (HD)	β-Phyllandrene (31.5%), α-terpinolene (22.5%), and 1,8-cineole (15.2)	[96]
*C. longa* L.	Vietnam	Leaf (HD)	α-Phellandrene (24.5%), 1,8-cineole (15.9%), *p*-cymene (13.2%) and β-pinene (8.9%)	[97]
*C. longa* L.	India	Leaf (HD)	Terpinolene (87.8%)	[58]
*C. longa* L.	India	Leaf (HD)	Myrcene (48.8%) and terpinolene (10.1%)	[58]
*C. mangga* Valeton and Zijp	Pahang, Malaysia	Rhizome (SD)	Caryophyllene oxide (18.7%) and caryophyllene (12.7%)	[28]
*C. mangga* Valeton and Zijp	Malaysia	Rhizome (HD)	Myrcene (46.5%) and β-pinene (14.6%)	[98]
*C. mangga* Valeton and Zijp	Penang, Malaysia	Rhizome (HD)	Myrcene (78.7%) and (*E*)-β-ocimene (5.1%)	[99]
*C. mangga* Valeton and Zijp	Malaysia	Rhizome (HD)	Myrcene (81.4%)	[31]
*C. nankunshanensis* N. Liu, X.B. Ye and Juan Chen	Huizhou, China	Fresh rhizome (SD)	Curdione (23.7%), germacrone (18.8%), 8,9-dehydro-9-formyl-cycloisolongifolene (10.7%), and velleral (6.1%)	[49]
*C. oligantha* Trimen	Badulla, Sri Lanka	Rhizome (HD)	Caryophyllene (15.1%), phytol (13.4), α-humulene (8.2%), γ-elemene (6.1%), and caryophyllene oxide (5.8%)	[13]
*C. phaeocaulis* Valeton	China	Rhizome (SD)	8,9-Dehydro-9-formyl-cycloisolongifolene (15.6–46.2%), germacrone (8.9–21.2%), curlone (0.8–20.2%), α-caryophyllene (0.1–11.0%), curzerene (0.6–9.8%), and β-elemene (0.6–5.4%)	[100]
*C. pierreana* Gagnep.	Vietnam	Flower (HD)	Isoborneol (27.3%), camphor (24.1%), isobornyl acetate (7.3%), camphene (6.7%), and α-pinene (5.1%)	[101]
*C. pseudomontana* J. Graham	Tamil Nadu, India	Rhizome (HD)	β-Elemenone (22.1%), pseudocumenol (20.7%), germacrone (15.2%), 2-(4-methoxyphenyl) *N*, *N*-trimethyl-1-pyrrolamine (13.1%), and (1,5 dimethyl-4-hexenyl)-4-methylbenzene (7.3%)	[102]
*C. purpurascens* Blume	Yogyakarta, Indonesia	Dried rhizome (HD)	Turmerone (13.5%), germacrone (13.2%), *ar*-turmerone (9.4%), germacrene-B (8.8%), curlone (6.2%), and curzerene (5.8%)	[103]
*C. rhabdota* Sirirugsa and M.F. Newman	Bangkok, Thailand	Fresh rhizome (HD)	Germacrone (24.4%), butyl butanoate (14.2%), *sec*-butyl butanoate (8.8%), camphene (7.0%), and germacrene B (6.3%)	[104]
*C. rubescens* Roxb.	Guangzhou, China	Fresh rhizome (SD)	Zerumbone (15.5%), *ar*-turmerone (13.8%), germacrone (13.5%), camphor (8.7%), and aromadendrene oxide (7.1%)	[49]
*C. sichuanensis* X.X. Chen	Chengdu, China	Fresh rhizome (SD)	Germacrone (28.1%), β-elemenone (10.7%), and isoaromadendrene epoxide (8.4%)	[49]
*C. sichuanensis* X.X. Chen	Sichuan, China	Dried rhizome (SD)	*ar*-Turmerone (43.5%), β-selinene (13.4%), δ-cadinene (13.2%), humulene oxide (8.0%), and curcumol (6.9%)	[50]
*C. sichuanensis* X.X. Chen	Sichuan, China	Rhizome (SD)	*epi*-Curzerenone (26.9%), germacrone (12.4%), isocurcumenol (9.7%), β-elemene (6.4%), and curzerene (6.2%)	[105]
*C. singulris* Gagnep.	Gia Lai, Vietnam	Fresh rhizome (SD)	Camphor (25.8%) and germacrone (8.0%)	[106]
*C. sylvatica* Valeton	Kerala, India	Rhizome (HD)	α-Fenchene (70.0%)	[17]
*C. trichosantha* Gagnep	Vietnam	Rhizome (HD)	Curdione (47.4%), curcumol (7.0%), and germacrone (6.1%)	[107]
*C. yunnanensis* N. Liu and S.J. Chen	Guangzhou, China	Fresh rhizome (SD)	Germacrone (13.5%), 8,9-dehydro-9-formyl-cycloisolongifolene (13.1%), dihydrocostunolide (12.3%), β-farnesene (7.5%), and aromadendrene oxide (7.4%)	[49]
*C. zanthorrhiza* Roxb.	Mustika Ratu Jakarta, Indonesia	Dry rhizome (SD)	α-Curcumene (64.8%) and camphor (6.0%)	[108]
*C. zanthorrhiza* Roxb.	Chiang Mai Province, Thailand	Rhizome (HD)	α-Terpinolene (24.9%), *p*-cymen-7-ol (12.2%), *p*-cymene (8.1%), and β-pinene (6.8%)	[2]
*C. zanthorrhiza* Roxb.	Kuala Selangor, Malaysia	Fresh rhizome (HD)	Xanthorrhizol (31.9%), β-curcumene (17.1%), *ar*-curcumene (13.2%), citronellyl pentanoate (5.7%), and camphor (5.4%)	[71]
*C. zanthorrhiza* Roxb.	Malaysia	Rhizome (HD)	Xanthorrhizol (44.5%)	[31]
*C. zedoaria* (Christm.) Roscoe	MaharajGanj, India	Rhizome (HD)	1,8-Cineole (18.5%), *p*-cymene (18.4%), and α-phellandrene (14.9%)	[37]
*C. zedoaria* (Christm.) Roscoe	Ruian, China	Rhizome (SD)	Curzerene (29.4%), curdione (19.6%), 1,8-cineole (9.7%), germacrone (9.2%), and β-elemene (8.1%)	[109]
*C. zedoaria* (Christm.) Roscoe	Changhwa, Taiwan	Dry rhizome (SD)	Epicurzerene (24.1%), curzerene (10.4%), and curdione (7.0%)	[7]
*C. zedoaria* (Christm.) Roscoe	China	Dry rhizome (HD)	Epicurzerene (46.6%), curdione (13.7%), and 5-isopropylidene-3,8-dimethyl-1(5*H*)-azulenone (9.2%)	[110]
*C. zedoaria* (Christm.) Roscoe	Kerala, India	Rhizome (HD)	Epicurzerenone (19.0%), *ar*-curcumene (12.1%), zingiberene (12.0%), β-sesquiphellandrene (9.8%), curzerene (8.0%), and germacrene B (6.0%).	[17]
*C. zedoaria* (Christm.) Roscoe	Gorakhpur, India	Rhizome (HD)	Curzerenone (31.6%), germacrone (10.8%) and camphor (10.3%)	[111]
*C. zedoaria* (Christm.) Roscoe	Colombo, Sri Lanka	Rhizome (HD)	Debromofiliforminol (31.5%), camphor (11.8%), aromadendrene (11.8%), benzofuran (8.8%), and germacrone (5.2%)	[13]
*C. zedoaria* (Christm.) Roscoe	Gorakhpur, India	Dry rhizome (HD)	Curzerene (31.6%), germacrone (10.8%), and camphor (10.3%)	[111]
*C. zedoaria* (Christm.) Roscoe	Northeast India	Rhizome (HD)	Curzerene (22.3%), 1,8-cineole (15.9%), and germacrone (9.0%)	[112]
*C. zedoaria* (Christm.) Roscoe	Kerala, India	Rhizome (HD)	1,8-Cineole (40.8%), curcumenene (18.7%), and camphor (10.2%)	[17]
*C. zedoaria* (Christm.) Roscoe	Kerala, India	Rhizome (HD)	1,8-Cineole (24.6%), β-sesquiphellandrene (21.5%), and elemenone (13.6%)	[17]
*C. zedoaria* (Christm.) Roscoe	Thailand	Rhizome (HD)	1,8-Cineol (37.6%) and curzerenone (13.7%)	[113]
*C. zedoaria* (Christm.) Roscoe	Shanghai, China	Commercial	Curzerene (26.5%), 1,8-cineole (12.0%), curcumol (9.0%), pyridine (8.0%), germacrone (7.9%), and β-elemene (7.4%)	[114]
*C. zedoaria* (Christm.) Roscoe	Lucknow, India	Leaf (HD)	α-Terpinyl acetate (8.4%), isoborneol (7.0%), dehydrocurdione (9.0%), and selina-4(15),7(11)-dien-8-one (9.4%)	[115]

HD = hydrodistillation; SD = steam distillation; SE = solvent extract; MTBE = methyl tert-butyl ether; SFE = supercritical fluid extraction; SPME = solid-phase microextraction; HSME = headspace solvent microextraction; PLE = pressurized liquid extraction.

**Table 2:** Biological activities of different *Curcuma* essential oils.

*Curcuma* Essential Oil	Biological Activity	Reference
*C. longa* rhizome EO	Antihyperlipidemic (in vivo, high-fat diet-induced hyperlipidemia rats, and hyperlipidemic golden Syrian hamsters)	[75,163]
	Antidiabetic and hypoglycemic (in vivo, obese diabetic rats, ≥620 mg/kg/day)	[164]
	Antiobesity (in vivo, obese diabetic rats, ≥620 mg/kg/day)	[165]
	α-Glucosidase and α-amylase inhibitor	[96,166,167]
	Antioxidant (in vitro, DPPH assay, FRAP assay, superoxide anion assay, and metal chelating assay)	[50,74,168,169]
	Neuroprotective (in vivo, postmyocardial ischemia/reperfusion in rats)	[166,170,171,172]
	Antiplatelet and antithrombosis (in vivo, myocardial ischemia-reperfusion and thrombosis rat models, 500 mg/kg, p.o.)	[172,173,174]
	Cytotoxic (in vitro, KB, P388, PANC-1, B16, LNCaP and HeLa cells)	[23,74,175,176,177,178]
	Anti-inflammatory (in vitro)	[176,178,179,180,181]
	Antiarthritic and joint-protective (in vivo, i.p., animal model of rheumatoid arthritis)	[23,182]
	Hepatoprotective and antihepatotoxic (in vivo, acute ethanol-induced fatty liver in rats, 200 mg/kg)	[23,183]
	Antiatherosclerotic	[96] [184]
	Hypothermic	[81]
	Anxiolytic	[81]
	Anticonvulsant	[81]
	Spasmolytic	[185]
	Antifatty liver (in vivo, acute ethanol-induced fatty liver in rats, 200 mg/kg)	[186]
	Antimutagenic (in vitro)	[178,187]
	Sedative and anesthetic (in vivo, mouse model and fish)	[81,96]
	Antivenom (in vivo, mouse model, *Bothrops jararaca* and *Crotalus durissus* venom)	[188]
	Antibacterial (*Helicobacter pylori*, *Bacillus cereus*, *B. coagulans*, *B. subtilis*, *Staphylococcus aureus*, *Escherichia coli*, *Vibrio parahaemolyticus*, *Proteus mirabilis*, and *Pseudomonas aeruginosa*)	[189,190]
	Antifungal (*Aspergillus flavus*, *A. niger*, *A. parasiticum*, *Rhizoctonia solani*, *Helminthosporium oryzae*, *Trichoconis padwickii*, *Curvularia lunata*, *C. pallescens*, *C. trifolii*, *Fusarium verticillioides*, *F. moniliforme*, *F. oxysporum*, *Penicillium digitatum*, *Alternaria dianthi*, *Trichophyton longifusus* and *Colletotrichum falcatum*)	[23,77,189,191,192]
	Antiaflatoxigenic	[76]
	Insecticidal (*Odontotermes obesus*)	[37,193,194]
	Insect repellent	[194,195]
	Mosquitocidal (*Aedes aegypti* and *Anopheles quadrimaculatus*)	[194]
	Phytotoxic (*Avena fatua*, *Echinochloa crus-galli*, *Allium cepa* and *Phalaris minor*)	[189]
*C. longa* leaf EO	Cytotoxic (in vitro, Hs578T and PC-3 cells)	[94]
	Antibacterial	[89,94,194]
	Antifungal and antiaflatoxigenic	[89,94,194]
	Mosquitocidal	[89,94,194]
*C. zedoaria* rhizome EO	Antioxidant (in vitro, DPPH assay)	[7,23,111,196,197]
	Cytotoxic (in vitro, SiHa, SNU-1, HepG2, AGS, B16BL6, SMMC-7721, SKOV3, H1299 and HL-60 cells)	[7,110,114,198,199]
	Antiangiogenic (in vitro and in vivo)	[200]
	Antitumor (in vivo, hepatoma-transplanted rats)	[201,202,203]
	Hypoglycemic (in vivo, streptozotocin-induced hyperglycemic Wistar rats)	[204]
	Anti-gingivitis (in vivo, streptozotocin-induced hyperglycemic Wistar rats)	[14,204]
	Anti-inflammatory	[14]
	Antimicrobial (*Vibrio parahaemolyticus*, *Staphylococcus aureus*, *Bacillus cereus*, *Salmonella typhimurium* and *Pseudomonas aeruginosa*)	[110]
	Antifungal (*Colletotrichum falcatum*)	[37]
	Insecticidal (*Odontotermes obesus*)	[37]
	Larvicidal (*Anopheles dirus*, LC_50_= 29.69 ppm; *Aedes aegypti*, LC_50_= 31.87 ppm)	[129]
*C. aeruginosa* rhizome EO	Antiandrogenic (in vivo, patients with androgenic alopecia, 5% *w*/*w*)	[30]
	Antinociceptive	[15]
	Antipyretic	[15]
	Anti-inflammatory	[15]
	Hair regrowth stimulant (in vivo, bald males)	[205]
	Skin penetration enhancer (in vivo, androgenic alopecia patients)	[30]
	Axillary hair-growth suppressant (in vivo, randomized double-blinded trial, 1 and 5% *w*/*w* EO)	[206]
	Axillary skin-brightness enhancer (in vivo, randomized double-blinded trial, 1 and 5% *w*/*w* EO)	[206]
	Antibacterial (*Enterococcus faecalis*, MIC = 6.25 µg/mL; *Streptococcus mutans*, MIC= 15.63 µg/mL; *Staphylococcus aureus*, MIC= 125 µg/mL; *Bacillus cereus*, MIC = 125 µg/mL)	[29,207]
	Antifungal (*Candida albicans*, MIC= 250 µg/mL)	[2]
	Antioxidant (in vitro, DPPH assay, EC_50_ = 24.32 µg/mL)	[29]
*C. aromatica* rhizome EO	Anti-inflammatory (in vitro)	[47,49]
	Cytotoxic (in vitro, LNCaP, HepG2, NSCLC and B16 cells)	[47,49,201,208,209]
	Antiproliferative (in vitro, Hep-2 cells; in vivo, mouse model with hepatoma)	[210]
	Antitumor (in vivo, patients with primary liver cancer; rats with transplanted hepatoma; and mouse model)	[211,212,213]
	Chemoprotective and antifibrosis (in vivo, renal interstitial fibrosis rats, 100, 200 and 300 mg/kg BW, i.p.)	[214,215]
	Antioxidant (in vitro, DPPH assay, ABTS assay and β-carotene bleaching tests)	[47,50,54,147]
	Antiplatelet aggregation and antithrombotic (in vitro and in vivo)	[216]
	Antibacterial (*Staphylococcus aureus*, *Listeria monocytogenes*, *Bacillus subtilis*, *Pseudomonas aeruginosa*, *Salmonella typhimurium*, *Escherichia coli*)	[47,54,217]
	Antifungal (*Candida albicans*, *Saccharomyces cerevisiae*)	[47]
	Cardioprotective (in vivo, isoproterenol-induced acute myocardial ischemia rats)	[218]
	Antidiabetic	[51]
	Insecticidal (*Liposcelis bostrychophila*)	[56]
	Antimosquito (*Aedes aegypti*)	[52]
*C. aromatica* leaf EO	Antifungal (*Colletotrichum falcatum*)	[37]
	Insecticidal (*Odontotermes obesus*)	[37]
*C. phaeocaulis* rhizome EO	Antimicrobial (*Escherichia coli*, *Pseudomonas aeruginosa*, *Staphylococcus aureus*)	[100,219]
	Antifungal (*Candida albicans*; *Saccharomyces cerevisiae*)	[100,219]
	Antioxidant (*in vitro*, DPPH assay, IC_50_ = 2.17–22.36 µg/mL)	[100]
	Anti-inflammatory (in vivo, TPA-induced skin inflammation model)	[100]
	Cytotoxic (*in vitro*, LNCaP and B16 cells, IC_50_ = 20.36–79.44 µg/mL)	[100]
*C. zanthorrhiza* rhizome EO	Antiproliferative	[220]
	Anti-inflammatory (*in vitro*)	[141,221]
	Antidiuretic	[141]
	Hypotensive	[141]
	Antihepatotoxic	[141]
	Antioxidant	[141,146]
	Antibacterial (*Staphylococcus aureus*, ZOI = 11.53 ± 0.27 mm)	[2,141,146]
	Antifungal (*Candida albicans*, ZOI = 7.29 ± 0.17 mm)	[2,141]
	Analgesic (in vivo, mouse model)	[222]
	Antihyperlipidemic (in vivo, rats, 0.2% or 0.5%)	[108]
	Antiobesogenic (in vivo, obese rats)	[108]
	Hypoglycemic and hypotriglyceridemic (in vivo, diabetic rats)	[223,224]
	Larvicidal	[146]
*C. amada* rhizome EO	Analgesic	[157]
	Anti-inflammatory	[157]
	Antiplatelet	[157]
	Cytotoxic (U-87MG, IC_50_ = 4.92 µg/mL; SJRH30, IC_50_ = 7.13 µg/mL); RD, IC_50_ = 7.50 µg/mL)	[157,225,226]
	Antitumor (human glioblastoma multiforme cells both in vitro and in nude mice xenografts)	[227]
	Hypotriglyceridemic	[157]
	Antifungal (*Physalospora tucumanensis*, *Sclerotium rolfsii*, *Helminthosporium sacchari*, *Cephalosporium sacchari*)	[157,228]
	Hepatoprotective (in vivo, carbon tetrachloride-induced hepatotoxicity in male Wister rats)	[156]
	Antioxidant (in vitro, DPPH assay, FRAP assay and nitric oxide scavenging assay)	[156,229]
	Antibacterial (*Staphylococcus aureus*, *Escherichia coli*, *Klebsiella pneumoniae*, *Pseudomonas aeruginosa*, *Salmonella paratyphi*, *Vibrio cholera*, *Enterobacter aerogenes*, *Streptococcus pneumoniae*, *Bacillus subtilis*, *Bacillus cereus*, *Proteus mirabilis*, *Proteus vulgaris*, *Serratia marcescens*)	[156,229]
	Insect repellent and insecticidal (*Musca domestica*)	[230]
*C. mangga* rhizome EO	Antibacterial (*Staphylococcus aureus*, MIC= 1.2 µL/mL; *Bacillus cereus*, MIC= 11.1 µL/mL; *P. aeruginosa*, ZOI = 9.0 mm; *E. coli*, ZOI= 7.0 mm)	[28]
	Antifungal (*Candida albicans*, MIC= 3.7 µL/mL; *Cryptococcus neoformans*, MIC= 0.1 µL/mL)	[28]
*C. glans* rhizome EO	Antibacterial (*Staphylococcus aureus*, ZOI= 17.24 ± 0.07 mm)	[2]
	Antifungal (*C. albicans*, ZOI= 7.27 ± 0.17 mm)	[2]
*C. singularis* rhizome EO	Antibacterial (*Bacillus subtillis*, MIC= 100 µg/mL; *E. coli*, MIC= 200 µg/mL)	[106]
*C. alismatifolia* rhizome EO	Antioxidant (in vitro, DPPH and FRAP assays)	[36]
*C. angustifolia* rhizome EO	Antioxidant	[45]
*C. elata* rhizome EO	Antioxidant (in vitro, DPPH assay)	[49]
	Cytotoxic (in vitro, LNCaP, IC_50_ = 18.4 μg/mL; HepG2, IC_50_ = 167.75 μg/mL)	[49]
	Anti-inflammatory (in vivo, TPA-induced edema model)	[49]
*C. kwangsiensis* rhizome EO	Cytotoxic (in vitro, LNCaP, B16 and HepG2)	[49,63]
	Antitumor	[62,63]
	Antioxidant	[62,63]
	Anti-inflammatory	[62,63]
	Bactericidal	[62,63]
	Antifungal	[62,63]
	Antiviral	[62,63]
*C. yunnanensis* rhizome EO	Cytotoxic (in vitro, LNCaP, B16 and HepG2)	[49]
*C. nankunshanensis* rhizome EO	Cytotoxic (in vitro, LNCaP, B16 and HepG2)	[49]
	Anti-inflammatory (in vivo, TPA-induced edema model)	[49]
*C. sichuanensis* rhizome EO	Cytotoxic (in vitro, LNCaP, B16 and HepG2)	[49]
	Antioxidant (in vitro, DPPH assay, IC_50_= 4.52 μg/mL)	[49,50]
	Anti-inflammatory (in vivo, TPA-induced edema model)	[49]
*C. rubescens* rhizome EO	Cytotoxic (in vitro, LNCaP, B16 and HepG2)	[49]
	Antioxidant (in vitro, DPPH assay, IC_50_ = 22.32 μg/mL)	[49]
*C. purpurascens* rhizome EO	Cytotoxic (in vitro, HT-29, IC_50_ = 4.9 ± 0.4 μg/mL)	[103]

**Table 3:** Biological activities of key components of *Curcuma* essential oils.

Compound	Biological Activity	Reference
*ar*-Turmerone	Antiplatelet Aggregation	[174]
	Antimutagenic	[178]
	Hypoglycemic	[167]
	Anti-inflammatory	[71,242,243]
	Neuroprotective	[244]
	Cytotoxic and antiproliferative	[220,245,246,247,248]
	Chemopreventive	[249]
	Insect repellent	[120]
	Antivenom	[188]
	Antibacterial	[250]
	Antifungal	[251]
Curdione	Anticancer	[252]
	Anti-inflammatory	[253]
	Antibacterial	[72]
	Antifungal	[72]
1,8-Cineole	Antioxidant	[254,255]
	Anticarcinogenic	[256]
β-Caryophyllene	Antitumor	[125,257,258,259]
	Antileishmanial	[260]
	Antitrypanosomal	[261]
Myrcene	Antimutagenic	[262]
	Chemopreventive	[263]
	Antiproliferative	[264,265]
	Antioxidant	[266]
Germacrone	Anti-inflammatory	[131,267]
	Antiandrogenic	[137]
	Skin-penetration enhancer	[30]
	Antiproliferative	[268,269,270]
	Antitumor	[270]
	Antioxidant	[271]
	Antibacterial	[28,272]
Xanthorrhizol	Antioxidant	[273,274]
	Nephroprotective	[273]
	Neuroprotective	[273,274]
	Chemopreventive	[249]
	Hepatoprotective	[273,274]
	Estrogenic	[273,274]
	Antiproliferative	[274]
	Antitumor	[275]
	Anti-inflammatory	[71]
	Antibacterial	[273,274]
β-Elemene	Antiproliferative	[210,237]
	Antiangionenic	[276]
	Hepatoprotective	[277]
	Antitumor	[278,279]
Terpinolene	Antioxidant	[280]
	Anti-inflammatory	[125]
	Chemoprotective	[263]
8,9-Dehydro-9-formylcycloiso-longifolene	Antioxidant	[281]
	Anti-inflammatory	[199]
Curcumol	Anticancer	[282]
Curzerene	Antioxidant	[168]
	Anticancer	[283]
β-Sesquiphellandrene	Antioxidant	[168]
	Anticancer	[284]
*ar*-Curcumene	Antitumor	[248]
α-Phellandrene	Antioxidant	[285,286]
	Antinociceptive	[285,286]
	Anti-inflammatory	[285,286]

## Abbreviations

A549	human lung-cancer cells
ABTS	2,2’-azinodi (3-ethyl benz-thiazoline sulfonic acid) diammonium salt
AGS	human gastric adenocarcinoma cells
Akt	protein kinase B
ASTC-a-1	human lung-adenocarcinoma cells
B16	melanoma cells
B16BL6	mouse melanoma cells
B16F10	melanoma cells
Ca Ski	human cervical cancer
CD14	cluster of differentiation 14
COX	Cyclooxygenase
CRTO	curcumin-removed turmeric oleoresin
DPPH	2,2-diphenyl-1-picrylhydrazyl
EC_50_	half maximal effective concentration
EO	essential oil
FRAP	ferric-reducing/antioxidant power
GRAS	generally recognized as safe
H1299	human lung-cancer cells
HCT116	human colon-cancer cells
HD	hydrodistillation
HDL	high-density lipoprotein
HeLa	human cervical-adenocarcinoma cells
Hep-2	laryngeal-cancer cells
HepG2	human hepatoma cell line
HFD	high-fat diet
HL-60	human myeloid leukemia cells
Hs578T	breast-tumor cells
HSME	headspace solvent microextraction
HT-29	human colorectal-cancer cells
i.p.	intraperitoneal
IC_50_	median inhibitory concentration
IKK	IκB kinase
iNOS	inducible nitric oxide synthase
J774.2	murine macrophages
K-562	Human erythroleukemia cells
KB	human mouth epidermal carcinoma cells
L1210	mouse lymphocytic leukemia cells
LC_50_	median lethal concentration
LD_100_	absolute lethal dose
LD_50_	median lethal dose
LDL	low-density lipoprotein
LNCaP	human prostate acedocarcinoma cells
LPS	lipopolysaccharide
MCF-7	human breast-cancer cells
MDA-MB-231	human breast-cancer cells
MIC	Minimal inhibitory concentration
NDMA	*N*-nitrosodimethylamine
NF-κB	nuclear factor-kappa B
NG108-15	mouse neuroblastoma cells
NSCLC	non-small-cell lung carcinoma cells
p.o.	per os (oral administration)
P388	mouse leukemia cells
PANC-1	pancreatic-cancer cells
PC-3	prostate-tumor cells
PGE2	prostaglandin E2
PKC	protein kinase C
PLE	pressurized liquid extraction
ppm	parts per million
RAW 264.7	mouse macrophage cells
RBL-2H3	rat leukemia cells
SD	steam distillation
SE	solvent extract
SFE	supercritical fluid extraction
SiHa	human cervical-cancer cells
SKOV3	human ovarian-cancer cells
SMMC-7721	human hepatoma cells
SNU-1	colorectal-cancer cells
SPME	solid phase microextraction
THP-1	human monocytes
TNF-α	tumor necrosis factor-α
TPA	12-*O*-tetradecanoylphorbol-13-acetate
U-251	human glioblastoma cells
U-87	human glioblastoma cells
U937	human lymphoma
VEGF	vascular endothelial growth factor
ZOI	zone of inhibition

## References

[1] Ravindran P.N., Babu K.N., Shiva K.N. (2007). Botany and crop improvement of tumeric. Turmeric The Genus Curcuma.

[2] Akarchariya N., Sirilun S., Julsrigival J., Chansakaowa S. (2017). Chemical profiling and antimicrobial activity of essential oil from *Curcuma aeruginosa* Roxb., *Curcuma glans* K. Larsen & J. Mood and *Curcuma* cf. *xanthorrhiza* Roxb. collected in Thailand. Asian Pac. J. Trop. Biomed..

[3] Leong-Skornikova J., Newman M. (2015). Gingers of Cambodia, Laos & Vietnam.

[4] Chuakul W., Boonpleng A. (2003). Ethnomedical uses of Thai Zingiberaceous plant (1). Thai. J. Phytopharm..

[5] Basaka S., Sarma G.C., Rangan L. (2010). Ethnomedical uses of Zingiberaceous plants of Northeast India. J. Ethnopharmacol..

[6] Jayaprakasha G.K., Jagan L., Rao M., Sakariah K.K. (2005). Chemistry and biological activity of *Curcuma longa*. Trend Food Sci. Technol..

[7] Mau J., Lai E.Y.C., Wang N.P., Chen C.C., Chang C.H., Chyau C.C. (2003). Composition and antioxidant activity of the essential oil from *Curcuma zedoaria*. Food Chem..

[8] Lobo R., Prabhu K.S., Shirwaikar A. (2009). *Curcuma zedoaria* Rosc (white turmeric): A review of its chemical, pharmacological and ethnomedicinal properties. J. Pharm. Pharmacol..

[9] Itokawa H., Shi Q., Akiyama T., Morris-Natschke S.L., Lee K.H. (2008). Recent advances in the investigation of curcuminoids. Chin. Med..

[10] Sikha A., Harini A., Prakash H. (2015). Pharmacological activities of wild turmeric (*Curcuma aromatica* Salisb): A review. J. Pharmacogn. Phytochem..

[11] Afzal A., Oriqat G., Khan M.A., Jose J., Afzal M. (2013). Chemistry and biochemistry of terpenoids from *Curcuma* and related species. J. Biol. Act. Prod. Nat..

[12] Krup V., Prakash H.L., Harini A. (2013). Pharmacological activities of turmeric (*Curcuma longa* Linn): A review. J. Tradit. Med. Clin. Naturop..

[13] Herath H.M.I.C., Wiyasiriwardene T.D.C.M.K., Premakumara G.A.S. (2017). Comparative GC-MS analysis of all *Curcuma* species grown in Sri Lanka by multivariate test. Ruhunu J. Sci..

[14] Chen I.N., Chang C.C., Ng C.C., Wang C.Y., Shyu Y.T., Chang T.L. (2008). Antioxidant and antimicrobial activity of Zingiberaceae plants in Taiwan. Plants Food Hum. Nutr..

[15] Reanmongkol W., Subhadhirasakul S., Khaisombat N., Fuengnawakit P., Jantasila S., Khamjun A. (2006). Investigation the antinociceptive, antipyretic and anti-inflammatory activities of *Curcuma aeruginosa* Roxb. extracts in experimental animals. Songklanakarin J. Sci. Technol..

[16] Wilson B., Abraham G., Manju V.S., Mathew M., Vimala B., Sundaresan S., Nambisan B. (2005). Antimicrobial activity of *Curcuma zedoaria* and *Curcuma malabarica* tubers. J. Ethnopharmacol..

[17] Angel G.R., Menon N., Vimala B., Nambisan B. (2014). Essential oil composition of eight starchy *Curcuma* species. Ind. Crops Prod..

[18] Sacchetti G., Maielti S., Muzzoli M., Scaglianti M., Manfredini S., Radice M., Bruni R. (2005). Comparative evaluation of 11 essential oils of different origin as functional antioxidants, antiradicals and antimicrobials in foods. Food Chem..

[19] Raut J.S., Karuppayil S.M. (2014). A status review on the medicinal properties of essential oils. Ind. Crops Prod..

[20] Weiss E.A. (2002). Spice Crops.

[21] Gopalan B., Goto M., Kodama A., Hirose T. (2000). Supercritical carbon dioxide extraction of turmeric (*Curcuma longa*). J. Agric. Food Chem..

[22] Jayaprakasha G.K., Negi P.S., Anandharamakrishnan C., Sakariah K.K. (2001). Chemical composition of turmeric oil—A byproduct from turmeric oleoresin industry and its inhibitory activity against different fungi. Z. Naturforsch. Sect. C J. Biosci..

[23] Singh G., Kapoor I.P.S., Singh P., De Heluani C.S., De Lampasona M.P., Catalan C.A.N. (2010). Comparative study of chemical composition and antioxidant activity of fresh and dry rhizomes of turmeric (*Curcuma longa* Linn.). Food Chem. Toxicol..

[24] Dosoky N.S. (2015). Isolation and Identification of Bioactive Compounds from *Conradina canescens* Gray. Ph.D. Dissertation.

[25] Sanghamitra N., Sujata M., Nagar K. (2015). Differential effect of soil and environment on metabolic expression of turmeric (*Curcuma longa* cv. Roma). Indian J. Exp. Biol..

[26] Srinivasan V., Thankamani C.K., Dinesh R., Kandiannan K., Zachariah T.J., Leela N.K., Hamza S., Shajina O., Ansha O. (2016). Nutrient management systems in turmeric: Effects on soil quality, rhizome yield and quality. Ind. Crops Prod..

[27] Burt S. (2004). Essential oils: Their antibacterial properties and potential applications in foods—A review. Int. J. Food Microbiol..

[28] Kamazeri T.S.A.T., Samah O.A., Taher M., Susanti D., Qaralleh H. (2012). Antimicrobial activity and essential oils of *Curcuma aeruginosa*, *Curcuma mangga*, and *Zingiber cassumunar* from Malaysia. Asian Pac. J. Trop. Med..

[29] Theanphong O., Mingvanish W., Kirdmanee C. (2015). Chemical constituents and biological activities of essential oil from *Curcuma aeruginosa* Roxb. rhizome. Bull. Heal. Sci. Technol..

[30] Srivilai J., Waranuch N., Tangsumranjit A., Khorana N., Ingkaninan K. (2018). Germacrone and sesquiterpene-enriched extracts from *Curcuma aeruginosa* Roxb. increase skin penetration of minoxidil, a hair growth promoter. Drug Deliv. Transl. Res..

[31] Jantan I.B., Ahmad A.S., Ali N.A.M., Ahmad A.R., Ibrahim H. (1999). Chemical composition of the rhizome oils of four *Curcuma* species from Malaysia. J. Essent. Oil Res..

[32] Sirat M.H., Jamil S., Hussain J. (1998). Essential oil of *Curcuma aeruginosa* Roxb. from Malaysia. J. Essent. Oil Res..

[33] Simoh S., Zainal A. (2015). Chemical profiling of *Curcuma aeruginosa* Roxb. rhizome using different techniques of solvent extraction. Asian Pac. J. Trop. Biomed..

[34] Jirovetz L., Buchbauer G., Puschmann C., Shafi M.P., Geetha Nambiar M.K. (2000). Essential oil analysis of *Curcuma aeruginosa* Roxb. leaves from South India. J. Essent. Oil Res..

[35] Dũng N.X., Tuyêt N.T.B., Leclercq P.A. (1995). Characterization of the leaf oil of *Curcuma aeruginosa* Roxb. from Vietnam. J. Essent. Oil Res..

[36] Theanphong O. (2017). Chemical constituents and antioxidant activities of essential oils from roots and rhizomes of *Curcuma alismatifolia* Gagnap. from Thailand. J. Appl. Sci..

[37] Singh G., Singh O.P., Maurya S. (2002). Chemical and biocidal investigations on essential oils of some Indian *Curcuma* species. Prog. Cryst. Growth Charact. Mater..

[38] Padalia R.C., Verma R.S., Sundaresan V., Chauhan A., Chanotiya C.S., Yadav A. (2013). Volatile terpenoid compositions of leaf and rhizome of *Curcuma amada* Roxb. from northern India. J. Essent. Oil Res..

[39] Choudhury S.N., Rabha L.C., Kanjilal P.B., Ghosh A.C., Leclercq P.A. (1996). Essential oil of *Curcuma amada* Roxb. from northeastern India. J. Essent. Oil Res..

[40] Mustafa A., Ali M., Khan N.Z. (2005). Volatile oil constituents of the fresh rhizomes of *Curcuma amada* Roxb. J. Essent. Oil Res..

[41] Rao A.S., Rajanikanth B., Seshadri R. (1989). Volatile aroma components of *Curcuma amada* Roxb. J. Agric. Food Chem..

[42] Srivastava A.K., Srivastava S.K., Shah N.C. (2001). Constituents of the rhizome essential oil of *Curcuma amada* Roxb. from India. J. Essent. Oil Res..

[43] Srivastava S., Chitranshi N., Srivastava S., Dan M., Rawat A.K.S., Pushpangadan P. (2006). Pharmacognostic evaluation of *Curcuma aeruginosa* Roxb. Nat. Prod. Sci..

[44] Thongkhwan P., Chaibunga T., Kwanboonjan H., Theanphong O. (2017). Essential oil constituents of the fresh root and rhizome of *Curcuma angustifolia* Roxb. from Thailand. Bull. Heal. Sci. Technol..

[45] Jena S., Ray A., Banerjee A., Sahoo A., Nasim N., Sahoo S., Kar B., Patnaik J., Panda P.C., Nayak S. (2017). Chemical composition and antioxidant activity of essential oil from leaves and rhizomes of *Curcuma angustifolia* Roxb. Nat. Prod. Res..

[46] Bordoloi A.K., Sperkova J., Leclercq P.A. (1999). Essential oils of *Curcuma aromatica* Salisb. from northeast India. J. Essent. Oil Res..

[47] Xiang H., Zhang L., Yang Z., Chen F., Zheng X., Liu X. (2017). Chemical compositions, antioxidative, antimicrobial, anti-inflammatory and antitumor activities of *Curcuma aromatica* Salisb. essential oils. Ind. Crops Prod..

[48] Choudhury S.N., Ghosh A.C., Saikia M., Choudhury M., Leclercq P.A. (1996). Volatile constituents of the aerial and underground parts of *Curcuma aromatica* Salisb from India. J. Essent. Oil Res..

[49] Xiang H., Zhang L., Xi L., Yang Y., Wang X., Lei D., Zheng X., Liu X. (2018). Phytochemical profiles and bioactivities of essential oils extracted from seven *Curcuma* herbs. Ind. Crops Prod..

[50] Tsai S.Y., Huang S.J., Chyau C.C., Tsai C.H., Weng C.C., Mau J.L. (2011). Composition and antioxidant properties of essential oils from *Curcuma* rhizome. Asian J. Arts Sci..

[51] Nampoothiri S.V., Philip R.M., Kankangi S., Kiran C.R., Menon A.N. (2015). Essential oil composition, α-amylase inhibition and antiglycation potential of *Curcuma aromatica* Salisb. J. Essent. Oil Bear. Plants.

[52] Choochote W., Chaiyasit D., Kanjanapothi D., Rattanachanpichai E., Jitpakdi A., Tuetun B., Pitasawat B. (2005). Chemical composition and anti-mosquito potential of rhizome extract and volatile oil derived from *Curcuma aromatica* against *Aedes aegypti* (Diptera: Culicidae). J. Vector. Ecol..

[53] Cao J., Qi M., Zhang Y., Zhou S., Shao Q., Fu R. (2006). Analysis of volatile compounds in *Curcuma wenyujin* Y.H. Chen et C. Ling by headspace solvent microextraction-gas chromatography-mass spectrometry. Anal. Chim. Acta.

[54] Al-Reza S.M., Rahman A., Sattar M.A., Rahman M.O., Fida H.M. (2010). Essential oil composition and antioxidant activities of *Curcuma aromatica* Salisb. Food Chem. Toxicol..

[55] Rameshkumar K.B., Sheeja D.B.A., Nair M.S., George V. (2015). *Curcuma ecalcarata*—New natural source of pinocembrin and piperitenone. Nat. Prod. Res..

[56] Liu Z.L., Zhao N.N., Liu C.M., Zhou L., Du S.S. (2012). Identification of insecticidal constituents of the essential oil of *Curcuma wenyujin* rhizomes active against *Liposcelis bostrychophila* Badonnel. Molecules.

[57] Pandey A.K., Chowdhury A.R. (2003). Volatile constituents of the rhizome oil of *Curcuma caesia* Roxb. from central India. Flavor Fragr. J..

[58] Behura S., Srivastava V.K. (2004). Essential oils of leaves of *Curcuma* species. J. Essent. Oil Res..

[59] Raj G., Baby S., Dan M., Thaha A.R.M., Sethuraman M.G., George V. (2008). Volatile constituents from the rhizomes of *Curcuma haritha* Mangaly and Sabu from southern India. Flavour Fragr. J..

[60] Dũng N., Truong P.X., Ky P.T., Leclercq P.A. (1997). Volatile constituents of the leaf, stem, rhizome, root and flower oils of *Curcuma harmandii* Gagnep. from Vietnam. J. Essent. Oil Res..

[61] Malek S.N., Seng C.K., Zakaria Z., Ali N.A., Ibrahim H., Jalil M.N. (2006). The essential oil of *Curcuma inodora* aff. Blatter from Malaysia. J. Essent. Oil Res..

[62] Zeng J.H., Xu G.B., Chen X. (2009). Application of the chromatographic fingerprint for quality control of essential oil from GuangXi *Curcuma kwangsiensis*. Med. Chem. Res..

[63] Zhang L., Yang Z., Huang Z., Zhao M., Li P., Zhou W., Zhang K., Zheng X., Lin L., Tang J. (2017). Variation in essential oil and bioactive compounds of *Curcuma kwangsiensis* collected from natural habitats. Chem. Biodivers..

[64] Naveen Kumar K., Venkataramana M., Allen J.A., Chandranayaka S., Murali H.S., Batra H.V. (2016). Role of *Curcuma longa* L. essential oil in controlling the growth and zearalenone production of *Fusarium graminearum*. LWT-Food Sci. Technol..

[65] Chatterjee S., Variyar P.S., Gholap A.S., Bongirwar D.R. (2000). Effect of γ-irradiation on the volatile oil constituents of turmeric (*Curcuma longa*). Food Res. Int..

[66] Awasthi P.K., Dixit S.C. (2009). Chemical composition of *Curcuma longa* leaves and rhizome oil from the plains of northern India. J. Young Pharm..

[67] Kutti Gounder D., Lingamallu J. (2012). Comparison of chemical composition and antioxidant potential of volatile oil from fresh, dried and cured turmeric (*Curcuma longa*) rhizomes. Ind. Crops Prod..

[68] Raina V.K., Syamsundar S.K. (2005). Srivastava, K.V. Rhizome and leaf oil composition of *Curcuma longa* from the lower Himalayan region of northern India. J. Essent. Oil Res..

[69] Singh G., Maurya S., Catalan C.A.N., De Lampasona M.P. (2005). Chemical, antifungal, insecticidal and antioxidant studies on *Curcuma longa* essential oil and its oleoresin. Indian Perfum..

[70] Leela N.K., Tava A., Shafi P.M., John S.P., Chempakami B. (2002). Chemical Composition of essential oils of turmeric (*Curcuma longa* L.). Acta Pharm..

[71] Jantan I., Saputri F.C., Qaisar M.N., Buang F. (2012). Correlation between chemical composition of *Curcuma domestica* and *Curcuma xanthorrhiza* and their antioxidant effect on human low-density lipoprotein oxidation. Evid. Based Complement. Altern. Med..

[72] Naz S., Ilyas S., Parveen Z., Javed S. (2010). Chemical analysis of essential oils from turmeric (*Curcuma longa*) rhizome through GC-MS. Asian J. Chem..

[73] Naz S., Ilyas S., Jabeen S., Parveen Z. (2011). Composition and antibacterial activity of the essential oil from the rhizome of turmeric (*Curcuma longa* L.). Asian J. Chem..

[74] Zhang L., Yang Z., Chen F., Su P., Chen D., Pan W., Fang Y., Dong C., Zheng X., Du Z. (2017). Composition and bioactivity assessment of essential oils of *Curcuma longa* L. collected in China. Ind. Crops Prod..

[75] Ling J., Wei B., Lv G., Ji H., Li S. (2012). Anti-hyperlipidaemic and antioxidant effects of turmeric oil in hyperlipidaemic rats. Food Chem..

[76] Ferreira F.D., Kemmelmeier C., Arrotéia C.C., Da Costa C.L., Mallmann C.A., Janeiro V., Ferreira F.M.D., Mossini S.A.G., Silva E.L., Machinski M. (2013). Inhibitory effect of the essential oil of *Curcuma longa* L. and curcumin on aflatoxin production by *Aspergillus flavus* Link. Food Chem..

[77] Avanço G.B., Ferreira F.D., Bomfim N.S., Andréia de Souza Rodrigues dos Santos P., Peralta R.M., Brugnari T., Mallmann C.A., de Abreu Filho B.A., Mikcha J.M.G., Machinski M. (2017). *Curcuma longa* L. essential oil composition, antioxidant effect, and effect on *Fusarium verticillioides* and fumonisin production. Food Control.

[78] Braga M.E.M., Leal P.F., Carvalho J.E., Meireles M.A.A. (2003). Comparison of yield, composition and antioxidant activity of turmeric (*Curcuma longa* L.) extracts obtained using various techniques. J. Agric. Food Chem..

[79] Asghari G., Mostajeran A., Shebli M. (2009). Curcuminoid and essential oil components of turmeric at different stages of growth cultivated in Iran. Res. Pharm. Sci..

[80] Marongiu B., Porcedda S., Caredda A., Gioannis B.D., Piras A. (2002). Supercritical CO_2_ extraction of curcumin and essential oil from the rhizomes of turmeric (*Curcuma longa* L.). J. Essent. Oil Bear. Plants.

[81] Oyemitan I.A., Elusiyan C.A., Onifade A.O., Akanmu M.A., Oyedeji A.O., McDonald A.G. (2017). Neuropharmacological profile and chemical analysis of fresh rhizome essential oil of *Curcuma longa* (turmeric) cultivated in southwest Nigeria. Toxicol. Rep..

[82] Gardini F., Belletti N., Ndagijimana M., Guerzoni M.E., Tchoumbougnang F., Zollo P.H.A., Micci C., Lanciotti R., Kamdem S.L.S. (2009). Composition of four essential oils obtained from plants from Cameroon, and their bactericidal and bacteriostatic activity against *Listeria monocytogenes*, *Salmonella enteritidis* and *Staphylococcus aureus*. Afr. J. Microbiol. Res..

[83] Sharma R.K., Mishra B.P., Sharma T.C., Bordloi A.K., Pathak M.G., Leclercq P.A. (1997). Essential oil of *Curcuma longa* L. from Bhutan. J. Essent. Oil Res..

[84] Chane-Ming J., Vera R., Chalchat J.-C., Cabassu P. (2002). Chemical composition of essential oils from rhizomes, leaves and flowers of *Curcuma longa* L. from Reunion Island. J. Essent. Oil Res..

[85] Usman L.A., Hamid A.A., George O.C., Ameen O.M., Muhammad N.O., Zubair M.F., Lawal A. (2009). Chemical composition of rhizome essential oil of *Curcuma longa* L. growing in north central Nigeria. World J. Chem..

[86] Raina V.K., Srivastava S.K., Jain N., Ahmad A., Syamasundar K.V., Aggarwal K.K. (2002). Essential oil composition of *Curcuma longa* L. cv. Roma from the plains of northern India. Flavour Fragr. J..

[87] Martins A.P., Salgueiro L., Gonçalves M.J., Da Cunha A.P., Vila R., Canigueral S., Mazzoni V., Tomi F., Casanova J. (2001). Essential oil composition and antimicrobial activity of three zingiberaceae from S. Tomé e Principe. Planta Med..

[88] Sirat H.M., Jamil S., Rahman A.A. (1997). Rhizome oil of *Curcuma ochrorhiza* Val. J. Essent. Oil Res..

[89] Sindhu S., Chempakam B., Leela N.K., Suseela Bhai R. (2011). Chemoprevention by essential oil of turmeric leaves (*Curcuma longa* L.) on the growth of *Aspergillus flavus* and aflatoxin production. Food Chem. Toxicol..

[90] Garg S.N., Mengi N., Patra N.K., Charles R., Kumar S. (2002). Chemical examination of the leaf essential oil of *Curcuma longa* L. from the north Indian plains. Flavour Fragr. J..

[91] Oguntimein B.O., Weyerstahl P., Marschall-Weyerstahl H. (1990). Essential oil of *Curcuma longa* L. leaves. Flavour Fragr. J..

[92] Priya R., Prathapan A., Raghu K.G., Nirmala Menon A. (2012). Chemical composition and in vitro antioxidative potential of essential oil isolated from *Curcuma longa* L. leaves. Asian Pac. J. Trop. Biomed..

[93] Pande C., Chanotiya C.S. (2006). Constituents of the leaf oil of *Curcuma longa* L. from Uttaranchal. J. Essent. Oil Res..

[94] Essien E., Newby J., Walker T., Setzer W., Ekundayo O. (2015). Chemotaxonomic characterization and in-vitro antimicrobial and cytotoxic activities of the leaf essential oil of *Curcuma longa* grown in southern Nigeria. Medicines.

[95] Zaibunnisa A.H., Norashikin S., Mamot S., Osman H. (2009). An experimental design approach for the extraction of volatile compounds from turmeric leaves (*Curcuma domestica*) using pressurised liquid extraction (PLE). LWT-Food Sci. Technol..

[96] Saccol E.M.H., Londero É.P., Bressan C.A., Salbego J., Gressler L.T., Silva L.V.F., Mourão R.H.V., Oliveira R.B., Llesuy S.F., Baldisserotto B. (2017). Oxidative and biochemical responses in *Brycon amazonicus* anesthetized and sedated with *Myrcia sylvatica* (G. Mey.) DC. and *Curcuma longa* L. essential oils. Vet. Anaesth. Analg..

[97] Dũng N.X., Tuyecirct N.T.B., Leclercq P.A. (1995). Constituents of the leaf oil of *Curcuma domestica* L. from Vietnam. J. Essent. Oil Res..

[98] Wahab I.R., Blagojević P.D., Radulović N.S., Boylan F. (2011). Volatiles of *Curcuma mangga* Val. & Zijp (Zingiberaceae) from Malaysia. Chem. Biodivers..

[99] Wong K.C., Chong T.C., Chee S.G. (1999). Essential oil of *Curcumma mangga* Val. and Van Zijp. rhizome. J. Essent. Oil Res..

[100] Zhang L., Yang Z., Wei J., Su P., Pan W., Zheng X., Zhang K., Lin L., Tang J., Fang Y. (2017). Essential oil composition and bioactivity variation in wild-growing populations of *Curcuma phaeocaulis* Valeton collected from China. Ind. Crops Prod..

[101] Dũng N.X., Tuyêt N.T.B., Van Khlên P., Barthel A., Leclercq P.A. (1998). Chemical composition of the flower oil of *Curcuma pierreana* Gagnep. from Vietnam. J. Essent. Oil Res..

[102] Muniyappan N., Nagarajan N.S. (2014). Green synthesis of gold nanoparticles using *Curcuma pseudomontana* essential oil, its biological activity and cytotoxicity against human ductal breast carcinoma cells T47D. J. Environ. Chem. Eng..

[103] Hong S.L., Lee G.S., Syed Abdul Rahman S.N., Ahmed Hamdi O.A., Awang K., Aznam Nugroho N., Abd Malek S.N. (2014). Essential oil content of the rhizome of *Curcuma purpurascens* Bl. (Temu Tis) and its antiproliferative effect on selected human carcinoma cell lines. Sci. World J..

[104] Theanphong O., Jenjittikul T., Mingvanish W. (2015). The rhizome oil of *Curcuma rhabdota* Sirirugsa & M.F. Newman from Thailand. Plant Science: International Proceedings.

[105] Zhou X., Li Z., Liang G., Zhu J., Wang D., Cai Z. (2007). Analysis of volatile components of *Curcuma sichuanensis* X. X. Chen by gas chromatography-mass spectrometry. J. Pharm. Biomed. Anal..

[106] Cuong N.M., Ha V.T., Khanh P.N., Van D.T., Cuong T.D., Huong T.T., Thuy D.T.T., Nhan N.T., Hanh N.P., Toan T.Q. (2017). Chemical compositions and antimicrobial activity of essential oil from the rhizomes of *Curcuma singularis* growing in Vietnam. Am. J. Essent. Oils Nat. Prod..

[107] Ky P.T., Van De Ven L.J.M., Leclercq P.A., Dũng N.X. (1994). Volatile constituents of the essential oil of *Curcuma trichosantha* Gagnep. from Vietnam. J. Essent. Oil Res..

[108] Yasni S., Imaizumi K., Sin K., Sugano M., Nonaka G. (1994). Sidik Identification of an active principle in essential oils and hexane-soluble fractions of *Curcuma xanthorrhiza* Roxb. showing triglyceride-lowering action in rats. Food Chem. Toxicol..

[109] Zhou L., Zhang K., Li J., Cui X., Wang A., Huang S., Zheng S., Lu Y., Chen W. (2013). Inhibition of vascular endothelial growth factor-mediated angiogenesis involved in reproductive toxicity induced by sesquiterpenoids of *Curcuma zedoaria* in rats. Reprod. Toxicol..

[110] Lai E.Y.C., Chyau C.-C., Mau J.-L., Chen C.-C., Lai Y.-J., Shih C.-F., Lin L.-L. (2004). Antimicrobial activity and cytotoxicity of the essential oil of *Curcuma zedoaria*. Am. J. Chin. Med..

[111] Singh P., Singh S., Kapoor I.P.S., Singh G., Isidorov V., Szczepaniak L. (2013). Chemical composition and antioxidant activities of essential oil and oleoresins from *Curcuma zedoaria* rhizomes, part-74. Food Biosci..

[112] Purkayastha J., Nath S.C., Klinkby N. (2006). Essential oil of the rhizome of *Curcuma zedoaria* (Christm.) Rosc. native to northeast India. J. Essent. Oil Res..

[113] Jarikasem S., Thubthimthed S., Chawananoraseth K., Suntorntanasat T. (2005). Essential oils from three *Curcuma* species collected in Thailand. Acta Hortic..

[114] Shi H., Tan B., Ji G., Lu L., Cao A., Shi S., Xie J. (2013). Zedoary oil (Ezhu You) inhibits proliferation of AGS cells. Chin. Med..

[115] Garg S.N., Naquvi A.A., Bansal R.P., Bahl J.R., Kumar S. (2005). Chemical composition of the essential oil from the leaves of *Curcuma zedoaria* Rosc. of Indian origin. J. Essent. Oil Res..

[116] Dahal K.R., Idris S., De Guzman C.C., Siemonsma J.S. (1999). Plant Resources of South-East Asia No 13. Spices.

[117] Zachariah T.J., Babu K.N. (1992). Effect of storage of fresh turmeric rhizome on oleoresin and curcumin contents. J. Spice Arom. Crop.

[118] Jayashree E., John Zachariah T. (2016). Processing of turmeric (*Curcuma longa*) by different curing methods and its effect on quality. Indian J. Agric. Sci..

[119] Govindarajan V.S. (1980). Turmeric-Chemistry, technology and quality. CRC Crit. Rev. Food Sci. Nutr..

[120] Helen C.F., Su R.H., Ghulam J. (1982). Isolation, purification and characterization of insect repellents from *Curcuma longa* L.. J. Agric. Food Chem..

[121] Srimal R.C. (1997). Turmeric: A brief review of medicinal properties. Fitoterapia.

[122] Narayana D.B.A., Brindavanam N.B., Dobriyal R.M., Katuyar K.C. (2000). Indian spices: An overview with special reference to neutraceuticals. J. Med. Aromat. Plant Sci..

[123] Paranjpe P. (2001). Herbs for Beauty.

[124] Chempakam B., Parthasarathy V.A., Parthasarathy V.A., Chempakam B., Zachariah T.J. (2008). Chemistry of Spices.

[125] Tisserand R., Young R. (2014). Essential Oil Safety.

[126] Balakrishnan K.V., Ravindra P.N., Nirmal Babu K., Sivaraman K. (2007). Post harvest technology and processing of turmeric. Turmeric, The Genus Curcuma.

[127] Parveen Z., Nawaz S., Siddique S., Shahzad K. (2013). Composition and antimicrobial activity of the essential oil from leaves of *Curcuma longa* L. Kasur variety. Indian J. Pharm. Sci..

[128] Bansal R.P., Bahl J.R., Garg S.N., Naqvi A.A., Sushil K., Kumar S. (2002). Differential chemical composition of the essential oils of the shoot organs, rhizomes and rhizoids in the turmeric *Curcuma longa* grown in Indo-Gangetic plains. Pharm. Biol..

[129] Pitasawat B., Champakaew D., Choochote W., Jitpakdi A., Chaithong U., Kanjanapothi D., Rattanachanpichai E., Tippawangkosol P., Riyong D., Tuetun B. (2007). Aromatic plant-derived essential oil: An alternative larvicide for mosquito control. Fitoterapia.

[130] Seo W.G., Hwang J.C., Kang S.K., Jin U.H., Suh S.J., Moon S.K., Kim C.H. (2005). Suppressive effect of Zedoariae rhizome on pulmonary metastasis of B16 melanoma cells. J. Ethnopharmacol..

[131] Makabe H., Maru N., Kuwabara A., Kamo T., Hirota M. (2006). Anti-inflammatory sesquiterpenes from *Curcuma zedoaria*. Nat. Prod. Res..

[132] Navarro Dde F., De Souza M.M., Neto R.A., Golin V., Niero R., Yunes R.A., Delle Monache F., Filho V.C. (2002). Phytochemical analysis and analgesic properties of *Curcuma zedoaria* grown in Brazil. Phytomedicine.

[133] Matsuda H., Tewtrakul S., Morikawa T., Nakamura A., Yoshikawa M. (2004). Anti-allergic principles from Thai zedoary: Structural requirements of curcuminoids for inhibition of degranulation and effect on the release of TNF-alpha and IL-4 in RBL-2H3 cells. Bioorg. Med. Chem..

[134] Ansari M.H., Ahmad S. (1991). Screening of some medicinal plants for antiamoebic action. Fitoterapia.

[135] Thaina P., Tungcharoena P., Wongnawaa M., Reanmongkol W., Subhadhirasakul S. (2009). Uterine relaxant effects of *Curcuma aeruginosa* Roxb. rhizome extracts. J. Ethnopharmacol..

[136] Sookchot T. (2005). Chemotaxonomy of Medicinal and Auspicious Plants in Zingiberaceae Sold at Ban Thum, Chiang Dao District, Chiang Mai Province. Master’s Thesis.

[137] Suphrom N., Pumthong G., Khorana N., Waranuch N., Limpeanchob N., Ingkaninan K. (2012). Anti-androgenic effect of sesquiterpenoids isolated from the rhizomes of *Curcuma aeruginosa* Roxb. Fitoterapia.

[138] Nanda Y., Singson N., Rao A.N. (2013). Ethnomedicinal plants of Thadou tribe of Manipur (India)-1. Pleione.

[139] Neamsuvan O., Tuwaemaengae T., Bensulong F., Asae A., Mosamae K. (2012). A survey of folk remedies for gastrointestinal tract diseases from Thailand’s three southern border provinces. J. Ethnopharmacol..

[140] Choudhury D., Ghosal M., Das A.P., Mandal P. (2013). Development of single node cutting propagation techniques and evaluation of antioxidant activities of *Curcuma aeruginosa* Roxburgh rhizome. Int. J. Pharm. Pharm. Sci..

[141] Salleh N.M., Ismail S., Ab Halim M. (2016). Effects of *Curcuma xanthorrhiza* extracts and their constituents on phase II drug-metabolizing enzymes activity. Pharmacognosy Res..

[142] Hsu H.Y., Chen Y.P., Shen S.J., Hsu C.S., Chen C.C., Chang H.C. (1986). Oriental Materia Medica, A Concise Guide.

[143] Perry M.L. (1980). Medicinal Plants of East and South East Asia, Attributed Properties and Uses.

[144] Mary H.P.A., Susheela G.K., Jayasree S., Nizzy A.M., Rajagopal B., Jeeva S. (2012). Phytochemical characterization and antimicrobial activity of *Curcuma xanthorrhiza* Roxb. Asian Pac. J. Trop. Biomed..

[145] Salea R., Widjojokusumo E., Veriansyah B., Tjandrawinata R.R. (2014). Optimizing oil and xanthorrhizol extraction from *Curcuma xanthorrhiza* Roxb. rhizome by supercritical carbon dioxide. J. Food Sci. Technol..

[146] Sukari M.A.H., Saad S.M., Lajis N.H., Rahman M., Muse R., Yusuf U.K., Riyanto S. (2007). Chemical constituents and bioactivity of *Curcuma aeruginosa* Roxb. Nat. Prod. Sci..

[147] Li S.Y., Li S.P. (2009). Antioxidant activities of essential oil of *Curcuma longa* and *Curcuma wenyujin*. Int. J. Essent. Oil Ther..

[148] Shi J.H., Li C.Z., Liu D.L. (1981). Experimental research on the pharmacology of *Curcuma aromatica* volatile oil. Zhongyao Tongbao.

[149] Chadha Y.R. (2001). The Wealth of India. A Dictionary of Indian Raw Materials and Industrial Products.

[150] Chopra R.N., Nayar S.L., Chopra I.C. (1956). Glossary of Indian Medicinal Plants.

[151] Kim J.K., Jo C., Hwang H.J., Park H.J., Kim Y.J., Byun M.W. (2006). Color improvement by irradiation of *Curcuma aromatica* extract for industrial application. Radiat. Phys. Chem..

[152] Mao Q.Q., Zhen H., Zhong X.M., Feng C.R., Pan A.J., Li Z.Y., Ip S.P., Che C.T. (2010). Effects of SYJN, a Chinese herbal formula, on chronic unpredictable stress-induced changes in behavior and brain BDNF in rats. J. Ethnopharmacol..

[153] Wang L.Y., Zhang M., Zhang C.F., Wang Z.T. (2008). Diaryl derivatives from the root tuber of *Curcuma longa*. Biochem. Syst. Ecol..

[154] Wu D.L. (2015). Zingiberaceae Resource of China.

[155] Alonso-Amelot M.E. (2016). Multitargeted bioactive materials of plants in the *Curcuma* genus and related compounds: Recent advances. Studi. Nat. Prod. Chem..

[156] Varadarajan R., Mathew M.C.R., Souprayan S. (2018). Hepatoprotective efficacy of ethanolic extracts of rhizome *Curcuma amada* Roxb. in experimental rats. Ann. Plant Sci..

[157] Policegoudra R.S., Aradhya S.M., Singh L. (2011). Mango ginger (*Curcuma amada* Roxb.)—A promising spice for phytochemicals and biological activities. J. Biosci..

[158] Mohanta P.K., Rout S.D., Sahu H.K. (2006). Ethnomedicinal plant resources of Similipal Biosphere Reserve, Orissa, India. Zoo’s Print J..

[159] Kunwar R.M., Shrestha K.P., Bussmann R.W. (2010). Traditional herbal medicine in far-west Nepal: A pharmacological appraisal. J. Ethnobiol. Ethnomed..

[160] Rokaya M.B., Munzbergova Z., Timsina B. (2010). Ethnobotanical study of medicinal plants from the Humla district of western Nepal. J. Ethnopharmacol..

[161] Devi L.R., Rana V.S., Devi S.I., Verdeguer M., Blázquez M.A. (2012). Chemical composition and antimicrobial activity of the essential oil of *Curcuma leucorhiza* Roxb. J. Essent. Oil Res..

[162] Lakshmi S., Padmaja G., Remani P. (2011). Antitumour effects of isocurcumenol isolated from *Curcuma zedoaria* rhizomes on human and murine cancer cells. Int. J. Med. Chem..

[163] Singh V., Jain M., Misra A., Khanna V., Rana M., Prakash P., Malasoni R., Dwivedi A.K., Dikshit M., Barthwal M.K. (2013). Curcuma oil ameliorates hyperlipidaemia and associated deleterious effects in golden Syrian hamsters. Br. J. Nutr..

[164] Nishiyama T., Mae T., Kishida H., Tsukagawa M., Mimaki Y., Kuroda M., Sashida Y., Takahashi K., Kawada T., Nakagawa K. (2005). Curcuminoids and sesquiterpenoids in turmeric (*Curcuma longa* L.) suppress an increase in blood glucose level in type 2 diabetic KK-Ay mice. J. Agric. Food Chem..

[165] Honda S., Aoki F., Tanaka H., Kishida H., Nishiyama T., Okada S., Matsumoto I., Abe K., Mae T. (2006). Effects of ingested turmeric oleoresin on glucose and lipid metabolisms in obese diabetic mice: A DNA microarray study. J. Agric. Food Chem..

[166] Omosa L.K., Midiwo J.O., Kuete V. (2012). Curcuma longa.

[167] Lekshmi P.C., Arimboor R., Indulekha P.S., Menon A.N. (2012). Turmeric (*Curcuma longa* L.) volatile oil inhibits key enzymes linked to type 2 diabetes. Int. J. Food Sci. Nutr..

[168] Zhao J., Zhang J.S., Yang B., Lv G.P., Li S.P. (2010). Free radical scavenging activity and characterization of sesquiterpenoids in four species of *Curcuma* using a TLC bioautography assay and GC–MS analysis. Molecules.

[169] Himaja M., Ranjitha A., Ramana M.V., Anand M., Karigar A. (2010). Phytochemical screening and antioxidant activity of rhizome part *Curcuma zedoaria*. Int. J. Res. Ayurveda Pharm..

[170] Dohare P., Garg P., Sharma U., Jagannathan N.R., Ray M. (2008). Neuroprotective efficacy and therapeutic window of *Curcuma* oil: In rat embolic stroke model. BMC Complement. Altern. Med..

[171] Rathore P., Dohare P., Varma S., Ray A., Sharma U., Jaganathanan N.R., Ray M. (2008). *Curcuma* oil: Reduces early accumulation of oxidative product and is anti-apoptogenic in transient focal ischemia in rat brain. Neurochem. Res..

[172] Manhas A., Khanna V., Prakash P., Goyal D., Malasoni R., Naqvi A., Dwivedi A.K., Dikshit M., Jagavelu K. (2014). *Curcuma* oil reduces endothelial cell-mediated inflammation in postmyocardial ischemia/reperfusion in rats. J. Cardiovasc. Pharmacol..

[173] Prakash P., Misra A., Surin W.R., Jain M., Bhatta R.S., Pal R., Raj K., Barthwal M.K., Dikshit M. (2011). Anti-platelet effects of *Curcuma* oil in experimental models of myocardial ischemia-reperfusion and thrombosis. Thromb. Res..

[174] Lee H.S. (2006). Antiplatelet property of *Curcuma longa* L. rhizome-derived *ar*-turmerone. Bioresour. Technol..

[175] Jacob J.N. (2016). Comparative studies in relation to the structure and biochemical properties of the active compounds in the volatile and nonvolatile rractions of turmeric (*C. longa*) and ginger (*Z. officinale*). Stud. Nat. Prod. Chem..

[176] Arora R., Basu N., Kapoor V., Jain A. (1971). Anti-inflammatory studies on *Curcuma longa* (turmeric). Indian J. Med. Res..

[177] Manosroi J., Dhumtanom P., Manosroi A. (2006). Anti-proliferative activity of essential oil extracted from Thai medicinal plants on KB and P388 cell lines. Cancer Lett..

[178] Jayaprakasha G.K., Jena B.S., Negi P.S., Sakariah K.K. (2002). Evaluation of antioxidant activities and antimutagenicity of turmeric oil: A byproduct from curcumin production. Z. Naturforsch..

[179] Hastak K., Lubri N., Jakhi S.D., More C., John A., Ghaisas S.D., Bhide S.V. (1997). Effect of turmeric oil and turmeric oleoresin on cytogenetic damage in patients suffering from oral submucous fibrosis. Cancer Lett..

[180] Joshi J., Ghaisas S., Vaidya A., Vaidya R., Kamat D.V., Bhagwat A.N., Bhide S. (2003). Early human safety study of turmeric oil (*Curcuma longa* oil) administered orally in healthy volunteers. J. Assoc. Physicians India.

[181] Lantz R.C., Chen G.J., Solyom A.M., Jolad S.D., Timmermann B.N. (2005). The effect of turmeric extracts on inflammatory mediator production. Phytomedicine.

[182] Funk J.L., Frye J.B., Oyarzo J.N., Zhang H., Barbara N. (2010). Anti-arthritic effects and toxicity of the essential oils of turmeric (*Curcuma longa* L.). J. Agric. Food Chem..

[183] Kiso Y., Suzuki Y., Watarable N. (1983). Antihepatotoxic principles of *Curcuma longa* rhizomes. Planta Med..

[184] Singh V., Rana M., Jain M., Singh N., Naqvi A., Malasoni R., Dwivedi A.K., Dikshit M., Barthwal M.K. (2015). *Curcuma* oil attenuates accelerated atherosclerosis and macrophage foam-cell formation by modulating genes involved in plaque stability, lipid homeostasis and inflammation. Br. J. Nutr..

[185] Shafreen R.B., Lubinska M., Różańska A., Dymerski T., Namieśnik J., Katrich E., Gorinstein S. (2018). Human serum interactions with phenolic and aroma substances of Kaffir (*Citrus hystrix*) and Key lime (*Citrus aurantifolia*) juices. J. Lumin..

[186] Nwozo S.O., Osunmadewa D.A., Oyinloye B.E. (2014). Anti-fatty liver effects of oils from *Zingiber officinale* and *Curcuma longa* on ethanol-induced fatty liver in rats. J. Integr. Med..

[187] Palasa K., Scsikaran B., Krishna T.P., Krishnaswamy K. (1992). Effect of turmeric on urinary mutagens in smokers. Mutagenesis.

[188] Ferreira L.A.F., Henriques O.B., Andreoni A.A.S., Vital G.R.F., Campos M.M.C., Habermehl G.G., De Moraes V.L.G. (1992). Antivenom and biological effects of *ar*-turmerone isolated from *Curcuma longa* (Zingiberaceae). Toxicon.

[189] Fagodia S.K., Singh H.P., Batish D.R., Kohli R.K. (2017). Phytotoxicity and cytotoxicity of *Citrus aurantiifolia* essential oil and its major constituents: Limonene and citral. Ind. Crops Prod..

[190] Negi P.S., Jayaprakasha G.K., Jagan Mohan Rao L., Sakariah K.K. (1999). Antibacterial activity of turmeric oil: A byproduct from curcumin manufacture. J. Agric. Food Chem..

[191] Behura C., Ray P., Rathi C.C., Mishra R.K., Ramachandraiah O.S., Charyulu J.K. (2000). Antifungal activity of essential oil of *Curcuma longa* against five rice pathogens in vitro. J. Essent. Oil Bear. Plants.

[192] Apisariyakul A., Vanittanakom N., Buddhasukh D. (1995). Antifungal activity of turmeric oil extracted from *Curcuma longa* (Zingiberaceae). J. Ethnopharmacol..

[193] Fouad H.A., Da Camara C.A.G. (2017). Chemical composition and bioactivity of peel oils from *Citrus aurantiifolia* and *Citrus reticulata* and enantiomers of their major constituent against *Sitophilus zeamais* (Coleoptera: Curculionidae). J. Stored Prod. Res..

[194] Roth G.N., Chandra A., Nair M.G. (1998). Novel bioactivities of *Curcuma longa* constituents. J. Nat. Prod..

[195] Tawatsin A., Wratten S.D., Scott R.R., Thavara U., Techadamrongsin Y. (2001). Repellency of volatile oils from plants against three mosquito vectors. J. Vector Ecol..

[196] Sumathi S., Iswariya G.T., Sivaprabha B., Dharani B., Radha P., Padma P.R. (2013). Comparative study of radical scavenging activity and phytochemical analysis of fresh and dry rhizomes of *Curcuma zedoaria*. Int. J. Pharm. Sci. Res..

[197] Angel G.R., Vimala B., Bala N. (2012). Antioxidant and antimicrobial activity of essential oils from nine starchy *Curcuma* species. Int. J. Curr. Pharm. Res..

[198] Myoungae K. (2003). Cytotoxic activity of the extracts from *Curcuma zedoaria*. J. Toxicol. Environ. Health.

[199] Chen C., Chen Y., Hsi Y.T., Chang C.S., Huang L.F., Ho C.T., Way T.D., Kao J.Y. (2013). Chemical constituents and anticancer activity of *Curcuma zedoaria* Roscoe essential oil against non-small cell lung carcinoma cells *in vitro* and *in vivo*. J. Agric. Food Chem..

[200] Chen W., Lu Y., Gao M., Wu J., Wang A., Shi R. (2011). Anti-angiogenesis effect of essential oil from *Curcuma zedoaria* in vitro and in vivo. J. Ethnopharmacol..

[201] Cheng J.H., Chang G., Wu W.Y. (2001). A controlled clinical study between hepatic arterial infusion with embolized *Curcuma* aromatic oil and chemical drugs in treating primary liver cancer. Zhongguo Zhong Xi Yi Jie He Za Zhi.

[202] Zhou Y., Shen J., Xia L., Wang Y. (2015). *Curcuma zedoaria* (Berg.) Rosc. essential oil and paclitaxel synergistically enhance the apoptosis of SKOV3 cells. Mol. Med. Rep..

[203] Wu W.Y., Luo Y.J., Cheng J.H. (1998). Zedoary turmeric oil inhibits transplantal hepatoma in rat via hepatic artery perfusion. World Chin. J. Dig..

[204] Handajani J., Narissi D.H. (2015). The effects of *Curcuma zedoaria* oil on high blood sugar level and gingivitis. Dent. J..

[205] Pumthong G., Asawanonda P., Varothai S., Jariyasethavong V., Triwongwaranat D., Suthipinittharm P., Ingkaninan K., Leelapornpisit P., Waranuch N. (2012). *Curcuma aeruginosa*, a novel botanically derived 5-α-reductase inhibitor in the treatment of male-pattern baldness: A multicenter, randomized, double-blind, placebo-controlled study. J. Dermatolog. Treat..

[206] Srivilai J., Phimnuan P., Jaisabai J., Luangtoomma N., Waranuch N., Khorana N., Wisuitiprot W., Scholfield C.N., Champachaisri K., Ingkaninan K. (2017). *Curcuma aeruginosa* Roxb. essential oil slows hair-growth and lightens skin in axillae; a randomised, double blinded trial. Phytomedicine.

[207] Wahyuni W.T., Batubara I., Tambunan D.Y. (2017). Antibacterial and teeth biofilm degradation activity of *Curcuma aeruginosa* essential oil. J. Biol. Sci..

[208] Ma J.W., Tsao T.C., His Y.T., Lin Y.C., Chen Y., Chen Y., Ho C.T., Kao J.Y., Way T.D. (2016). Essential oil of *Curcuma aromatica* induces apoptosis in human non-small-cell lung carcinoma cells. J. Funct. Foods.

[209] Li Y., Wo J.M., Liu Q., Li X., Martin R.C. (2009). Chemoprotective effects of *Curcuma aromatica* on esophageal carcinogenesis. Ann. Surg. Oncol..

[210] Tao L., Zhou L., Zheng L., Yao M. (2006). Elemene displays anti-cancer ability on laryngeal cancer cells in vitro and in vivo. Cancer Chemother. Pharmacol..

[211] Wu W.Y., Luo Y.J., Cheng J.H., Chang G., Liu W.S., Li R.X. (1998). Therapeutic effect of *Curcuma aramatica* oil infused via hepatic artery against transplanted hepatoma in rats. Huaren Xiaohua Zazhi.

[212] Wu W.Y., Xu Q., Shi L.C., Zhang W.B. (2000). Inhibitory effects of *Curcuma aromatica* oil on proliferation of hepatoma in mice. World J. Gastroenterol..

[213] Cheng J.H., Wu W.Y., Liu W.S., Chang G., Liu Y.L., Yang Z.G., Li L.N., Zhou H. (1999). Treatment of 17 cases of patients with primary liver cancer with *Curcuma aromatica* oil infused via hepatic artery. Shijie Huaren Xiaohua Zazhi.

[214] Li Y., Shi X., Zhang J., Zhang X., Martin R.C.G. (2014). Hepatic protection and anticancer activity of *Curcuma*: A potential chemopreventive strategy against hepatocellular carcinoma. Int. J. Oncol..

[215] Zhao L., Zhang H., Yang Y., Zheng Y., Dong M., Wang Y., Bai G., Ye X., Yan Z., Gao H. (2014). Serum metabonomic analysis of protective effects of *Curcuma aromatica* oil on renal fibrosis rats. PLoS ONE.

[216] Xia Q., Wang X., Xu D.J., Chen X.H., Chen F.H. (2012). Inhibition of platelet aggregation by curdione from *Curcuma wenyujin* essential oil. Thromb. Res..

[217] Al-Reza S.M., Rahman A., Parvin T., Rahman M.M., Rahman M.S. (2011). Chemical composition and antibacterial activities of essential oil and organic extracts of *Curcuma aromatica* Salisb. J. Food Saf..

[218] Li Y., Feng J., Mo Y., Liu H., Yang B. (2016). Concordance between cardio-protective effect on isoproterenol-induced acute myocardial ischemia and phenolic content of different extracts of *Curcuma aromatica*. Pharm. Biol..

[219] Liu H.H., Wei Y., Cui J., Wang L.H., Liu S.G., Long Z.F. (2008). Antifungal activities of the extracts from *Curcuma phaeocaulis* against *Phoma wasabiae*. J. Sichuan Univ. (Nat. Sci. Ed.).

[220] Schmidt E., Ryabchenko B., Wanner J., Jäger W., Jirovetz L. (2015). Cytotoxic active constituents of essential oils of *Curcuma longa* and *Curcuma zanthorrhiza*. Nat. Prod. Commun..

[221] Ozaki Y. (1990). Antiinflammatory effect of *Curcuma xanthorrhiza* Roxb and its active principles. Chem. Pharm. Bull..

[222] Wicaksono A.J., Yuniarti N., Pramono S. (2015). Analgesic effect of combination of essential oil Curcuma xanthorriza Roxb. and its curcuminoids in mice. Trad. Med. J..

[223] Yasni S., Imaizumi K., Sugano M. (1991). Effects of an Indonesian medicinal plant, *Curcuma xanthorrhiza* Roxb. on the levels of serum glucose and triglyceride, fatty acid desaturation, and bile acid excretion in streptozotocin-induced diabetic rats. Agric. Biol. Chem..

[224] Yasni S., Imaizumi K., Nakamura M., Aimoto J., Sugano M. (1993). Effects of *Curcuma xanthorrhiza* Roxb. and curcuminoids on the level of serum and liver lipids, serum apolipoprotein A-I and lipogenic enzymes in rats. Food Chem. Toxicol..

[225] Ramachandran C., Quirin K.W., Escalon E.A., Lollett I.V., Melnick S.J. (2015). Therapeutic effect of supercritical CO_2_ extracts of *Curcuma* species with cancer drugs in rhabdomyosarcoma cell lines. Phytother. Res..

[226] Ramachandran C., Lollett I.V., Escalon E., Quirin K.W., Melnick S.J. (2015). Anticancer potential and mechanism of action of mango ginger (*Curcuma amada* Roxb.) supercritical CO_2_ extract in human glioblastoma cells. J. Evid. Based Complement. Altern. Med..

[227] Ramachandran C., Portalatin G.M., Prado A.M., Quirin K.W., Escalon E., Melnick S.J. (2017). In vivo antitumor effect of supercritical CO_2_ extract of mango ginger (*Curcuma amada* Roxb) in U-87MG human glioblastoma nude mice xenografts. J. Evid. Based Complement. Altern. Med..

[228] Kumar A., Singh L., Chomwal R., Sawal R. (2009). Anti-microbial potential of the rhizome extracts of *Curcuma amada* Roxb. J. Pharm. Res..

[229] George M., Britto S.J., Arulappan M.T., Marandi R.R., Kindo I., Dessy V.J. (2015). Phytochemical, antioxidant and antibacterial studies on the essential oil of the rhizome of *Curcuma amada* Roxb. Int. J. Curr. Res..

[230] Singh D., Singh A.K. (1991). Repellent and insecticidal properties of essential oils against housefly, *Musca domestica* L.. Int. J. Trop. Insect Sci..

[231] Dohare P., Garg P., Jain V., Nath C., Ray M. (2008). Dose dependence and therapeutic window for the neuroprotective effects of curcumin in thromboembolic model of rat. Behav. Brain Res..

[232] Li C., Li L., Luo J., Huang N. (1998). Effect of turmeric volatile oil on the respiratory tract. Zhongguo Zhong Yao Za Zhi..

[233] Murakami A., Furukawa I., Miyamoto S., Tanaka T., Ohigashi H. (2013). Curcumin combined with turmerones, essential oil components of turmeric, abolishes inflammation-associated mouse colon carcinogenesis. Biofactors.

[234] Xiao Y., Yang F.Q., Li S.P., Hu G., Lee S.M., Wang Y.T. (2008). Essential oil of *Curcuma wenyujin* induces apoptosis in human hepatoma cells. World J. Gastroenterol..

[235] Su M.Q., Zhou Y.R., Li C.Q., Wang Z., Wang Y.L., Shen B.Y., Dou J. (2018). Zedoary Turmeric oil induces senescence and apoptosis in human colon cancer HCT116 cells. Nat. Prod. Commun..

[236] Wang Y.F., Liu S.Q., Zhao J.H. (2006). Observation of therapeutic effect of compound zedoary turmeric oil duppositories for treating monilial vaginitis with pregnancy. Hebei Yi Yao.

[237] Yang H., Wang X.P., Yu L.L., Zheng S. (1996). The antitumor activity of elemene is associated with apoptosis. Zhonghua Zhongliu Zazhi.

[238] Jena S., Ray A., Sahoo A., Kar B., Panda P.C., Nayak S. (2016). Chemical constituents of leaf essential oil of *Curcuma angustifolia* Roxb. growing in eastern India. J. Essent. Oil Bear. Plants.

[239] Opdyke D.L.J. (1973). Monographs on fragrance raw materials. Food Cosmet. Toxicol..

[240] Zhou N.N., Mao X.J., Zhang J., Yang S.W., Zhang J.M. (2004). Pharmacological investigation on contraindication of *Curcuma zedoaria*. Chin. Arch. Tradit. Chin. Med..

[241] Kong Y.C., Xie J.X., But P.P.H. (1986). Fertility regulating agents from traditional Chinese medicine. J. Ethnopharmacol..

[242] Oh S., Han A., Park H.R., Jang E.J., Kim H.K., Jeong M.G., Song H., Park G.H., Seo E.K., Hwang E.S. (2014). Suppression of inflammatory cytokine production by *ar*-turmerone isolated from *Curcuma phacaulis* essential oil. Chem. Biodivers..

[243] Rana M., Reddy S.S., Maurya P., Singh V., Chaturvedi S., Kaur K., Agarwal H., Ahmad H., Naqvi A., Dwivedi A.K. (2015). Turmerone enriched standardized *Curcuma longa* extract alleviates LPS induced inflammation and cytokine production by regulating TLR4–IRAK1–ROS–MAPK–NFkB axis. J. Funct. Foods.

[244] Hucklenbroich J., Klein R., Neumaier B., Graf R., Fink G.R., Schroeter M., Rueger M.A. (2014). Aromatic turmerone induces neural stem cell proliferation in vitro and in vivo. Stem Cell Res. Ther..

[245] Paek S.H., Kim G.J., Jeong H.S., Yum S.K. (1996). Ar-turmerone and β-atlantone induce internucleosomal DNA fragmentation associated with programmed cell death in human myeloid leukemia HL-60 cells. Arch. Pharmacol. Res..

[246] Baik K.U., Jung S.H., Ahn B.Z. (1993). Recognition of pharmacophore of *ar*-turmerone for its anticancer activity. Arch. Pharmacol. Res..

[247] Ji M., Choi J., Lee J., Lee Y. (2004). Induction of apoptosis by *ar*-turmerone on various cell lines. Int. J. Mol. Med..

[248] Itokawa H., Hirayama F., Funakoshi K., Takeya K. (1985). Studies on the antitumor bisabolane sesquiterpenoids isolated from *Curcuma xanthorrhiza*. Chem. Pharmacol. Bull..

[249] Lee S.K., Hong C.H., Huh S.K., Kim S.S., Oh O.J., Min H.Y., Park K.K., Chung W.Y., Hwang J.K. (2002). Suppressive effect of natural sesquiterpenoids on inducible cyclooxygenase (COX-2) and nitric oxide synthase (iNOS) activity in mouse macrophage cells. J. Environ. Pathol. Toxicol. Oncol..

[250] Lee H.S. (2006). Antimicrobial properties of turmeric (*Curcuma longa* L.) rhizome-derived *ar*-turmerone and curcumin. Food Sci. Biotechnol..

[251] Dhingra O.D., Jham G.N., Barcelos R.C., Mendonça F.A., Ghiviriga I. (2007). Isolation and identification of the principal fungitoxic component of turmeric essential oil. J. Essent. Oil Res..

[252] Li J., Bian W.H., Wan J., Zhou J., Lin Y., Wang J.R., Wang Z.X., Shen Q., Wang K.M. (2014). Curdione inhibits proliferation of MCF-7 cells by inducing apoptosis. Asian Pac. J. Cancer Prev..

[253] Oh O.J., Min H.Y., Lee S.K. (2007). Inhibition of inducible prostaglandin E2 production and cyclooxygenase-2 expression by curdione from *Curcuma zedoaria*. Arch. Pharm. Res..

[254] Perry N.S., Houghton P.J., Sampson J., Theobald A.E., Hart S., Lis-Balchin M., Hoult J.R., Evans P., Jenner P., Milligan S. (2001). In-vitro activity of *S. lavandulaefolia* (Spanish sage) relevant to treatment of Alzheimer’s disease. J. Pharm. Pharmacol..

[255] Saito Y., Shiga A., Yoshida Y., Furuhashi T., Fujita Y., Niki E. (2004). Effects of a novel gaseous antioxidative system containing a rosemary extract on the oxidation induced by nitrogen dioxide and ultraviolet radiation. Biosci. Biotechnol. Biochem..

[256] Moteki H., Hibasami H., Yamada Y., Katsuzaki H., Imai K., Komiya T. (2002). Specific induction of apoptosis by 1,8-cineole in two human leukemia cell lines, but not a in human stomach cancer cell line. Oncol. Rep..

[257] Lampronti I., Saab A.M., Gambari R. (2006). Antiproliferative activity of essential oils derived from plants belonging to the Magnoliophyta division. Int. J. Oncol..

[258] Loizzo M.R., Tundis R., Menichini F., Saab A.M., Statti G.A., Menichini F. (2008). Antiproliferative effects of essential oils and their major constituents in human renal adenocarcinoma and amelanotic melanoma cells. Cell Prolif..

[259] Owolabi M.S., Ogundajo A.L., Dosoky N.S., Setzer W.N. (2013). The cytotoxic activity of *Annona muricata* leaf oil from Badagary, Nigeria. Am. J. Essent. Oils Nat. Prod..

[260] Soares D.C., Portella N.A., Ramos M.F.D.S., Siani A.C., Saraiva E.M. (2013). *trans*-Caryophyllene: An effective antileishmanial compound found in commercial copaiba oil (*Copaifera* spp.). Evid.-Based Complement. Altern. Med..

[261] Izumi E., Ueda-Nakamura T., Veiga V.F., Pinto A.C., Nakamura C.V. (2012). Terpenes from *Copaifera* demonstrated in vitro antiparasitic and synergic activity. J. Med. Chem..

[262] De-Oliveira A.C., Ribeiro-Pinto L.F., Paumgartten J.R. (1997). In vitro inhibition of CYP2B1 monooxygenase by β-myrcene and other monoterpenoid compounds. Toxicol. Lett..

[263] Sawamura M., Sun S.H., Ozaki K., Ishikawa J., Ukeda H. (1999). Inhibitory effects of *Citrus* essential oils and their components on the formation of *N*-nitrosodimethylamine. J. Agric. Food Chem..

[264] Chaouki W., Leger D.Y., Liagre B., Beneytout J.L., Hmamouchi M. (2009). Citral inhibits cell proliferation and induces apoptosis and cell cycle arrest in MCF-7 cells. Fundam Clin. Pharmacol..

[265] Dosoky N.S., Pokharel S.K., Setzer W.N. (2015). Leaf essential oil composition, antimicrobial; and cytotoxic activities of *Cleistocalyx operculatus* from Hetauda, Nepal. Am. J. Essent. Oils Nat. Prod..

[266] Mitić-Culafić D., Zegura B., Nikolić B., Vuković-Gacić B., Knezević-Vukcević J., Filipic M. (2009). Protective effect of linalool, myrcene and eucalyptol against *t*-butyl hydroperoxide induced genotoxicity in bacteria and cultured human cells. Food Chem. Toxicol..

[267] Hossain C.F., Al-Amin M., Sayem A.S.M., Siragee I.H., Tunan A.M., Hassan F., Kabir M.M., Sultana G.N.N. (2015). Antinociceptive principle from *Curcuma aeruginosa*. BMC Complement. Altern. Med..

[268] Zhong Z., Chen X., Tan W., Xu Z., Zhou K., Wu T., Cui L., Wang Y. (2011). Germacrone inhibits the proliferation of breast cancer cell lines by inducing cell cycle arrest and promoting apoptosis. Eur. J. Pharmacol..

[269] Liu B., Gao Y.Q., Wang X.M., Wang Y.C., Fu L.Q. (2014). Germacrone inhibits the proliferation of glioma cells by promoting apoptosis and inducing cell cycle arrest. Mol. Med. Rep..

[270] Liu Y., Wang W., Fang B., Ma F., Zheng Q., Deng P., He G. (2013). Anti-tumor effect of germacrone on human hepatoma cell lines through inducing G2/M cell cycle arrest and promoting apoptosis. Eur. J. Pharmacol..

[271] Hamdi O.A.A., Ye L.J., Kamarudin M.N.A., Hazni H., Paydar M., Looi C.Y., Shilpi J.A., Kadir H.A., Awang K. (2015). Neuroprotective and antioxidant constituents from *Curcuma zedoaria* rhizomes. Rec. Nat. Prod..

[272] Diastuti H., Syah Y.M., Juliawaty L.D., Singgih M. (2014). Antibacterial activity of germacrane type sesquiterpenes from *Curcuma heyneana* rhizomes. Indones. J. Chem..

[273] Kim S.H., Hong K.O., Hwang J.K., Park K.K. (2005). Xanthorrhizol has a potential to attenuate the high dose cisplatin-induced nephrotoxicity in mice. Food Chem. Toxicol..

[274] Oon S.F., Nallappan M., Tee T.T., Shohaimi S., Kassim N.K., Sa’ariwijaya M.S.F., Cheah Y.H. (2015). Xanthorrhizol: A review of its pharmacological activities and anticancer properties. Cancer Cell Int..

[275] Choi M.A., Kim S.H., Chung W.Y., Hwang J.K., Park K.K. (2005). Xanthorrhizol, a natural sesquiterpenoid from *Curcuma xanthorrhiza*, has an anti-metastatic potential in experimental mouse lung metastasis model. Biochem. Biophys. Res. Commun..

[276] Chen W., Lu Y., Wu J., Gao M., Wang A., Xu B. (2011). Beta-elemene inhibits melanoma growth and metastasis via suppressing vascular endothelial growth factor-mediated angiogenesis. Cancer Chemother. Pharmacol..

[277] Liu J., Zhang Z., Gao J., Xie J., Yang L., Hu S. (2011). Downregulation effects of beta-elemene on the levels of plasma endotoxin, serum TNF-alpha, and hepatic CD14 expression in rats with liver fibrosis. Front. Med..

[278] Li Q.Q., Wang G., Huang F., Banda M., Reed E. (2010). Antineoplastic effect of beta-elemene on prostate cancer cells and other types of solid tumour cells. J. Pharm. Pharmacol..

[279] Tan P., Zhong W., Cai W. (2000). Clinical study on treatment of 40 cases of malignant brain tumor by elemene emulsion injection. Zhongguo Zhong Xi Yi Jie He Za Zhi.

[280] Kim H.J., Chen F., Wu C., Wang X., Chung H.Y., Jin Z. (2004). Evaluation of antioxidant activity of Australian tea tree (*Melaleuca alternifolia*) oil and its components. J. Agric. Food Chem..

[281] Li J., Li F., Xu Y., Yang W., Qu L., Xiang Q., Liu C., Li D. (2013). Chemical composition and synergistic antioxidant activities of essential oils from *Atractylodes macrocephala* and *Astragalus membranaceus*. Nat. Prod. Commun..

[282] Zhang W., Wang Z., Chen T. (2011). Curcumol induces apoptosis via caspases independent mitochondrial pathway in human lung adenocarcinoma ASTC-a-1 cells. Med. Oncol..

[283] Hsu B. (1980). The use of herbs as anticancer agents. Am. J. Chin. Med..

[284] Tyagi A.K., Prasad S., Yuan W., Li S., Aggarwal B.B. (2015). Identification of a novel compound (β-sesquiphellandrene) from turmeric (*Curcuma longa*) with anticancer potential: Comparison with curcumin. Investig. New Drugs.

[285] Lima D.F., Brandão M.S., Moura J.B., Leitão J.M.R.S., Carvalho F.A.A., Miúra L.M.C.V. (2012). Antinociceptive activity of the monoterpene α-phellandrene in rodents: Possible mechanisms of action. J. Pharm. Pharmacol..

[286] Da Silva J.K.R., Maia J.G.S., Dosoky N.S., Setzer W.N. (2016). Antioxidant, antimicrobial, and cytotoxic properties of *Aniba parviflora* essential oils from the Amazon. Nat. Prod. Commun..

[287] *ar*-Turmerone. https://www.sigmaaldrich.com/catalog/product/sial/42258?lang=en&region=US.

[288] Ciftci O., Ozdemir I., Tanyildizi S., Yildiz S., Oguzturk H. (2011). Antioxidative effects of curcumin, β-myrcene and 1,8-cineole against 2,3,7,8-tetrachlorodibenzo-*p*-dioxin-induced oxidative stress in rats liver. Toxicol. Ind. Heal..

[289] Melis K., Janssens G., Bochner A. (1990). Accidental nasal eucalyptol and menthol instillation. Acta Clin. Belg. Suppl..

[290] Jenner P.M., Hagan E.C., Taylor J.M., Al E. (1964). Food flavorings and compounds of related structure I. Acute oral toxicity. Food Cosmet. Toxicol..

[291] Kubo I., Chaudhuri S.K., Kubo Y., Sanchez Y., Ogura T., Saito T., Ishikawa H., Haraguchi H. (1996). Cytotoxic and antioxidative sesquiterpenoids from *Heterotheca inuloides*. Planta Med..

[292] Da Silva S.L., Figueiredo P.M., Yano T. (2007). Chemotherapeutic potential of the volatile oils from *Zanthoxylum rhoifolium* Lam leaves. Eur. J. Pharmacol..

[293] Frosch P.J., Johansen J.D., Menné T., Pirker C., Rastogi S.C., Andersen K.E., Bruze M., Goossens A., Lepoittevin J.P., White I.R. (2002). Further important sensitizers in patients sensitive to fragrances. Contact Dermat..

[294] Matura M., Skold M., Borje A., Al E. (2005). Selected oxidized fragrance terpenes are common contact allergens. Contact Dermat..

[295] Di Sotto A., Evandri M.G., Mazzanti G. (2008). Antimutagenic and mutagenic activities of some terpenes in the bacterial reverse mutation assay. Mutat. Res..

[296] Paumgartten F.J., De-Carvalho R.R., Souza C.A., Madi K., Chahoud I. (1998). Study of the effects of beta-myrcene on rat fertility and general reproductive performance. Braz. J. Med. Biol. Res..

[297] National Toxicology Program (2010). NTP technical report on the toxicology and carcinogenesis studies of beta-myrcene (CAS No. 123-35-3) in F344/N rats and B6C3F1 mice (gavage studies). Natl. Toxicol. Progr. Tech. Rep. Ser..

[298] Grosse Y., Loomis D., Guyton K.Z., El Ghissassi F., Bouvard V., Benbrahim-Tallaa L., Mattock H., Straif K. (2017). Some chemicals that cause tumours of the urinary tract in rodents. Lancet Oncol..

[299] Beta-Myrcene. https://www.sigmaaldrich.com/catalog/product/sial/64643?lang=en&region=US.

[300] Germacrone. https://www.sigmaaldrich.com/catalog/product/sial/42924?lang=en&region=US.

[301] Xanthorrhizol. https://www.caymanchem.com/product/14668.

[302] Wang G., Li X., Huang F., Zhao J., Ding H., Cunningham C., Coad J.E., Flynn D.C., Reed E., Li Q.Q. (2005). Antitumor effect of beta-elemene in non-small-cell lung cancer cells is mediated via induction of cell cycle arrest and apoptotic cell death. Cell. Mol. Life Sci..

[303] Zou L., Liu W., Yu L. (2001). Beta-Elemene induces apoptosis of K562 leukemia cells. Zhongguo Zhong Xi Yi Jie He Za Zhi.

[304] Yan B., Zhou Y., Feng S., Lv C., Xiu L., Zhang Y., Shi J., Li Y., Wei P., Qin Z. (2013). β-Elemene-attenuated tumor angiogenesis by targeting Notch-1 in gastric cancer stem-like cells. Evid. Based. Complement. Altern. Med..

[305] Opdyke D.L.J. (1976). Monographs on fragrance raw materials. Food Cosmet. Toxicol..

[306] Hausen B.M., Reichling J., Harkenthal M. (1999). Degradation products of monoterpenes are the sensitizing agents in tea tree oil. Am. J. Contact Dermat..

[307] Ahn B.Z., Lee J.H. (1989). Cytotoxic and cytotoxicity-potentiating effects of the *Curcuma* root on L1210 cell. Korean J. Pharmacogn..

